# Ligand-Binding Sites in Vanilloid-Subtype TRP Channels

**DOI:** 10.3389/fphar.2022.900623

**Published:** 2022-05-16

**Authors:** Maria V. Yelshanskaya, Alexander I. Sobolevsky

**Affiliations:** Department of Biochemistry and Molecular Biophysics, Columbia University, New York, NY, United States

**Keywords:** TRP channels, ligand, agonist, inhibitor, antagonist, blocker, cryo-EM, X-ray crystallography

## Abstract

Vanilloid-subfamily TRP channels TRPV1-6 play important roles in various physiological processes and are implicated in numerous human diseases. Advances in structural biology, particularly the “resolution revolution” in cryo-EM, have led to breakthroughs in molecular characterization of TRPV channels. Structures with continuously improving resolution uncover atomic details of TRPV channel interactions with small molecules and protein-binding partners. Here, we provide a classification of structurally characterized binding sites in TRPV channels and discuss the progress that has been made by structural biology combined with mutagenesis, functional recordings, and molecular dynamics simulations toward understanding of the molecular mechanisms of ligand action. Given the similarity in structural architecture of TRP channels, 16 unique sites identified in TRPV channels may be shared between TRP channel subfamilies, although the chemical identity of a particular ligand will likely depend on the local amino-acid composition. The characterized binding sites and molecular mechanisms of ligand action create a diversity of druggable targets to aid in the design of new molecules for tuning TRP channel function in disease conditions.

## Introduction

Transient receptor potential (TRP) channels are regulated by a broad range of stimuli, including chemicals, temperature, mechanical stress, and membrane voltage, and are directly involved in sensory perception such as thermal perception, nociception, taste, olfaction, vision, hearing, and touch (Clapham, 2003). TRP channels are implicated in the pathogenesis of numerous human diseases, including various types of cancer, asthma, hypertension, osteoporosis, pancreatitis, cystitis, allergy, psoriasis, taste dysfunction, neuropathic and inflammatory pain, stroke, migraine, neurodegenerative disorders, and schizophrenia, and represent important drug targets (Nilius et al., 2007; Moran et al., 2011; Arbabian et al., 2020). This superfamily of cation-selective ion channels includes seven subfamilies: TRPV (vanilloid), TRPC (canonical), TRPM (melastatin), TRPN (NOMPC-like), TRPA (ankyrin), TRPP (polycystic), and TRPML (mucolipin). The TRPV subfamily contains six members, TRPV1-6. TRPV1 represents the founding member of the TRP channel superfamily, being the first mammalian TRP channel to be cloned (Caterina et al., 1997) and the first TRP channel to be resolved structurally (Cao et al., 2013; Liao et al., 2013). The first four members of the TRPV subfamily, TRPV1-4, represent temperature-activated TRP channels or thermo-TRPs (Islas, 2014; Voets, 2014; Arrigoni and Minor, 2018; Castillo et al., 2018; Lamas et al., 2019; Yuan, 2019). All thermo-TRPVs are activated by heat but in different temperature ranges, from warm for TRPV4 (>27°C) (Güler et al., 2002) and TRPV3 (>31°C) (Peier et al., 2002; Smith et al., 2002; Xu et al., 2002) to noxiously hot for TRPV1 (>43°C) (Caterina et al., 1997) and TRPV2 (>52°C) (Caterina et al., 1999). TRPV5 and TRPV6 are not activated by temperature. Instead, they are highly calcium-selective, representing calcium uptake channels in epithelial tissues (Hoenderop et al., 1999; Peng et al., 1999; Yelshanskaya et al., 2021; Khattar et al., 2022). Despite diverse contributions of TRPV subfamily members to physiology and disease, they share structural architecture, which in turn determines common ligand-binding sites for pharmacological interventions.

### TRPV Channel Structural Architecture

**FIGURE 1:**
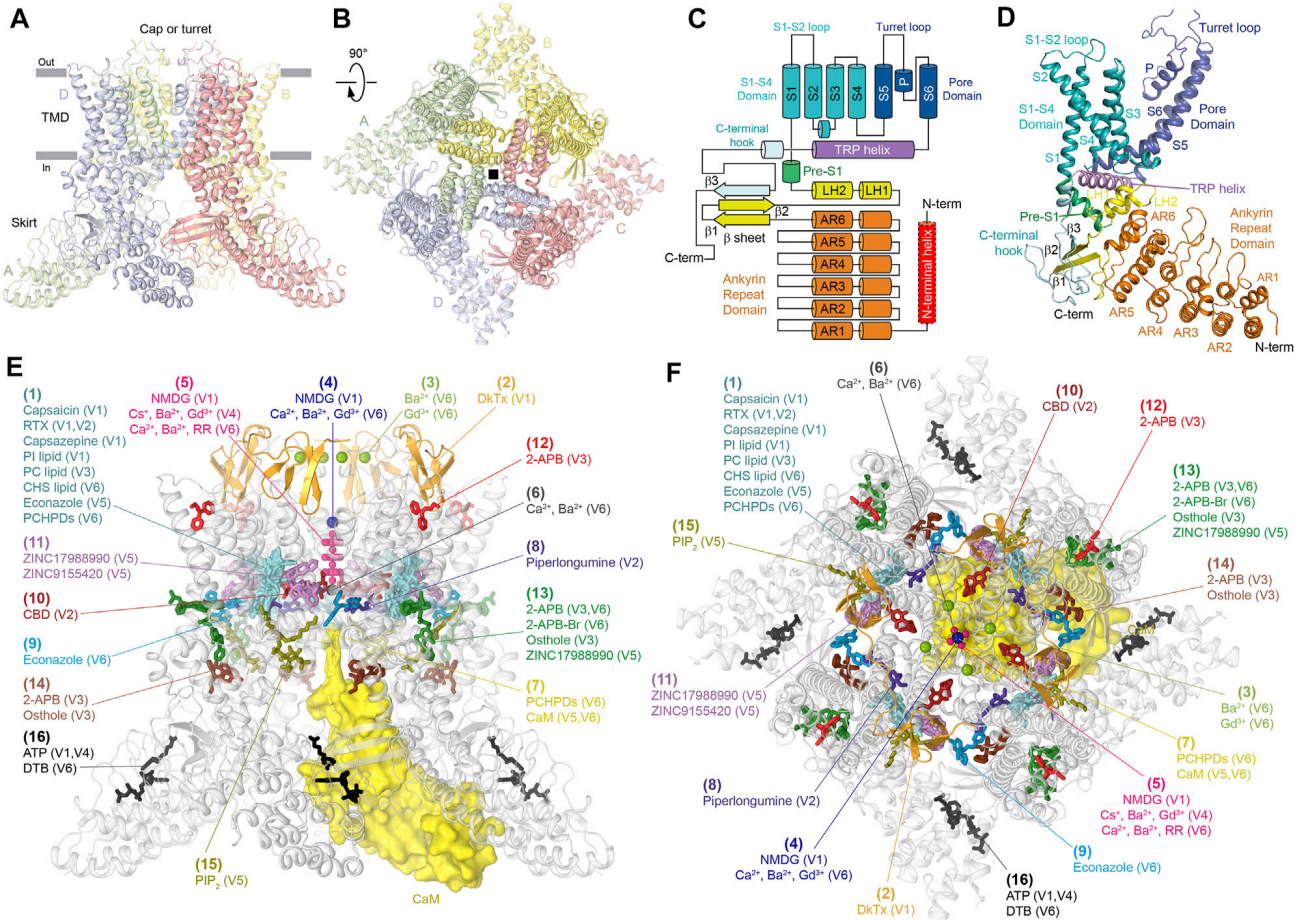
Vanilloid-subfamily TRP channel architecture and mapping of binding sites. **(A–B)** Side **(A)** and top **(B)** views of the TRPV1 tetramer (PDB ID: 7LQY) with each subunit shown in a different color. (C) Domain organization diagram of TRPV subunit. The N-terminal helix (red) is only present in TRPV5 and TRPV6. **(D)** Structure of the TRPV1 subunit with domains colored as in **(C)**. **(E)–(F)** Side **(E)** and top **(F)** views of the TRPV1 tetramer (gray) with 16 unique binding sites mapped by differently colored ligands.

Four 700 to 970 residue-long TRPV channel subunits assemble a four-fold symmetrical tetramer that includes two main compartments: a transmembrane domain (TMD) with a central ion channel pore and an intracellular skirt in which ankyrin repeat domains (ARDs) of four subunits connected by three-stranded β-sheets comprise walls enclosing a wide cavity underneath the ion channel (Figures 1A,B). TRPV1 also includes an extracellular cap or turret that forms a dome above the pore’s extracellular entrance, with four portals leading to the ion conductance pathway and is critical for channel function (Grandl et al., 2010; Yang et al., 2010; Nadezhdin et al., 2021a).

Each individual TRPV subunit (Figures 1C, D) has peripheral regions of N- and C-termini that are not resolved in crystal or cryo-EM structures and are likely disordered. The most N-terminally structured region in TRPV1-4 is the ARD. In TRPV5-6, the ARD is preceded by an additional structural element, the N-terminal helix, which is important for the assembly of these channels (Saotome et al., 2016). The ARD of TRPV channels contains six ankyrin repeats and is followed by the ARD-TMD linker domain that includes a β-hairpin (composed of β-strands, β1 and β2) and a helix-turn-helix motif (composed of linker helices, LH1 and LH2), resembling a seventh ankyrin repeat. The last C-terminal part of the ARD-TMD linker domain, the pre-S1 helix, connects this domain to the TMD, which crudely resembles the TMDs in voltage-gated potassium (Long et al., 2005; Long et al., 2007), sodium (Payandeh et al., 2011; Shen et al., 2017), and calcium (Wu et al., 2015) channels and includes six transmembrane helices (S1-S6) and a pore loop (P-loop) between S5 and S6. The first four transmembrane helices form a bundle to comprise the S1-S4 domain. In voltage-gated ion channels, the S1-S4 domain plays the role of a voltage sensor, in which positively charged arginine or lysine residues of S4 move relative to the membrane plane during activation and deactivation of these channels. In TRPV channels, S4 does not contain positively charged residues, thus explaining why these channels are not activated by membrane voltage.

S5, P-loop, and S6 comprise the pore domain. The pore domains of all four TRPV subunits assemble together in a four-fold symmetrical manner to form an ion conduction pathway or pore in the middle that connects two sides of the membrane, with the outside entry facing an extracellular vestibule and the cytoplasmic side entry facing the intracellular cavity. The extended region of each subunit re-entrant P-loop lines the extracellular part of the pore and contributes to a selectivity filter, which determines channel permeability and ion selectivity. The intracellular part of the pore is lined by the S6 helices with the narrow part of their bundle forming the gate. Between the gate and the selectivity filter is the pore’s central cavity, which is connected to the membrane environment surrounding the channel by four side portals between S5 and S6 of the neighboring subunits.

The S1-S4 and pore domains interact with each other in a domain-swapped arrangement, similar to classical voltage-gated channels but different from the non-swapped arrangement observed in CNG channels (Li et al., 2017), HCN channels (Lee and MacKinnon, 2017), and potassium channels such as Eag1 (Whicher and MacKinnon, 2016), Slo1 (Tao et al., 2017), and Slo2.2 (Hite and MacKinnon, 2017). Interestingly, the non-swapped arrangement was first discovered in the crystal structure of TRPV6 that had a single residue mutation L495Q in the TMD but showed nearly identical channel function to wild type (Saotome et al., 2016). The reversal of glutamine to leucine produced a remarkable conversion of TRPV6 to the natural domain-swapped architecture (Singh et al., 2017). A similar conversion can also happen as a result of deletions in the S4-S5 linker connecting the S1-S4 and pore domains (Singh et al., 2017), something that has likely happened in the course of evolution with CNG, HCN, Eag1, Slo1, and Slo2.2 channels (Whicher and MacKinnon, 2016; Hite and MacKinnon, 2017; Lee and MacKinnon, 2017; Li et al., 2017; Tao et al., 2017).

Following S6 is the amphipathic TRP helix, which runs parallel to the membrane and creates a hub for interactions between hydrophobic elements of the TMD and hydrophilic elements of the intracellular skirt. After the TRP helix, the polypeptide forms a loop structure named the C-terminal hook, which typically starts with a short α-helix and ends with a β-strand (β3) that tethers to the β-hairpin in the linker domain to create the three-stranded β-sheet. The latter, together with the C-terminal hook, participates in intersubunit interactions with the ARDs that glue the elements of the intracellular skirt together. In TRPV5-6, at the center of these interactions is the N-terminal helix, which is positioned as a pillar along the corners of the intracellular skirt (Saotome et al., 2016; Hughes et al., 2018a; Dang et al., 2019). Deletions of the N-terminal helix or mutations of its conserved residues in TRPV5 abolished or significantly impaired its Ca^2+^ uptake function (de Groot et al., 2011), suggesting that the intersubunit interactions are both structurally and functionally important. In TRPV1-3, which lack the N-terminal helix, intersubunit interactions are strengthened by the C-terminus, which wraps around the three-stranded β-sheet (Singh et al., 2018a; Zubcevic et al., 2018; Nadezhdin et al., 2021a).

The similarity in structural architecture of different TRPV channels allows generalized mapping and classification of ligand-binding sites that have been discovered in individual subfamily members (Figures 1E, F; Table 1). We identified 16 distinct binding sites, which form several groups based on contribution of specific structural elements: the vanilloid site (1); sites associated with ion channel pore, such as DkTx site (2); the permeant ions recruitment site in the extracellular vestibule (3); the extracellular pore entry site (4); the selectivity filter site (5); the central cavity site (6) and the intracellular pore entry site (7); the deep (8) and shallow (9) S4-S5 sites; the deep (10) and shallow (11) portal sites; the sites at the top (12) and base (13) of the S1-S4 helical bundle; the ARD-TMD linker (14) and S2-S3 (15) sites at the ARD–TMD interface; and the ARD sites (16).

**TABLE 1:** Ligand binding sites in TRPV channels.

Site	Ligand	Channel	Function	Affinity (μM) and references	Method: resolution	PDB IDs and references	Domains: residues
**(1)** Vanilloid site	Capsaicin	Rat TRPV1	Agonist	EC_50_ 0.2–1.9 Caterina et al. (1997); McIntyre et al. (2001); Vriens et al. (2009); Laursen et al. (2016)	Cryo-EM: 3.37–4.20 Å	3J5R, 7LPA, 7LPB, 7LPD, and 7LPE Cao et al. (2013); Kwon et al. (2021)	**S3:** Y511, S512, and L515
							**S4:** F543, T550, N551, and Y554
							**S4-S5:** I573
							**S6:** L662
		Squirrel TRPV1	Agonist	EC_50_ 0.53 ± 0.03 Laursen et al. (2016)	Cryo-EM:3.81 Å	7LR0 Nadezhdin et al. (2021a)	**S3:** S514, L517, and F518
							**S4:** F545, T552, N553, L555, and Y556
							**S5:** F593
							**S6:** L664 and L671
	Resiniferatoxin (RTX)	Rat TRPV1	Agonist	EC_50_ 0.007–0.1 Caterina et al. (1997); Szallasi et al. (1999); Vriens et al. (2009); Zhang et al. (2016)	Cryo-EM: 2.76–3.84 Å	3J5Q, 5IRX, 7L2L, 7L2M, 7L2N, 7L2O, 7L2V, 7L2W, 7L2X, 7MZ5, 7MZ7, 7MZ9, 7MZA, 7MZB, 7MZC, 7MZD, and 7MZE Cao et al. (2013); Gao et al. (2016); Zhang et al. (2021)	**S3:** Y511, S512, I514, and L515
							**S4:** A546, M547, T550, N551, L553, Y554, and R557
							**S4-S5:** A566, I569, and I573
							**S5:** F591
							**S6:** A665, I668, and L669
		Rabbit TRPV2	Agonist	–	X-ray:3.10 Å	6BWJ, 6OO3, 6OO4, 6OO5, and 6OO7 Zubcevic et al. (2018); Zubcevic et al. (2019a)	**S3:** Y469, S470, L473
					Cryo-EM:2.90–4.20 Å		**S4:** S501, V504, M505, T508, L511, and R515
							**S4-S5:** S524
							**S5:** I531, L539, and F545
							**S6:** L630
							**TRP:** Q661
		Squirrel TRPV1	Agonist	–	Cryo-EM 3.41 Å	7LQZ Nadezhdin et al. (2021a)	**S3:** Y513, S514, M516, L517, and F518
							**S4:** A548, M549, T552, N553, and Y556
							**S4-S5:** I575 and L579
							**S5:** F593
							**S6:** I670 and L671
	Capsazepine	Rat TRPV1	Competitive antagonist	IC_50_ 0.3 - 2.4 Bevan et al. (1992); Caterina et al. (1997); Szallasi et al. (1999)	Cryo-EM: 3.43 Å	5IS0 Gao et al. (2016)	**S3:** Y511, S512, and L515
							**S4:** M547, T550, N551, L553, and Y554
							**S4-S5:** E570
							**S5:** F591
							**S6:** L669
	Phosphatidyl-inositol (PI) lipid	Rat TRPV1	Unknown	–	Cryo-EM: 2.60–3.70 Å	5IRZ, 7L2H, 7L2I, 7L2J, 7L2P, 7L2R, 7L2S, 7L2T, 7L2U, 7LP9, 7LPC, and 7MZ6 Gao et al. (2016); Kwon et al. (2021); Zhang et al. (2021)	**LH2:** R409
							**S2-S3:** D511
							**S3:** S512, S514, Y513, and L517
							**S4:** A548, M549, T552, and L555
							**S4-S5:** E572, I575, and L576
							**S5:** F593
							**S6:** A667 and L671
							**TRP:** I698, L701, Q702, and I705
		Squirrel TRPV1	Unknown	–	Cryo-EM: 3.19 Å	7LQY Nadezhdin et al. (2021a)	**LH2:** R411 and H412
							**S2-S3:** D509 and Y511
							**S3:** S512 and L515
							**S4:** T550, L553, Y554, and R557
							**S4-S5:** E570
							**S6:** L669
							**TRP:** L699, Q700, and I703
	Phosphatidyl-choline (PC) lipid	Mouse TRPV3	Inhibitor	–	Cryo-EM: 1.98–3.42 Å	6LGP, 7MIJ, 7MIK, 7MIM, and 7MIN Shimada et al. (2020); Nadezhdin et al. (2021b)	**S2-S3:** L517, S518, and D519
							**S3:** W521, F522, and F524
							**S4:** A556, A560, L563, and R567
							**S4-S5:** S576, V577, I579, Q580, and L584
							**S5:** F601
							**TRP:** Q695
	Cholesteryl hemisuccinate (CHS) lipid	Human TRPV6	Unknown	–	Cryo-EM: 2.43–3.26 Å	7K4A, 7S88, 7S89, 7S8B, and 7S8C Bhardwaj et al. (2020) Neuberger et al. (2021a)	**S3:** P424 and F425
							**S4:** S455, V459, C463, and M466
							**S4-S5:** T479, I480, I482, and Q483
							**S6:** I557, T558, A561, and I565
							**TRP:** Q596
	Econazole	Rabbit TRPV5	Inhibitor	IC_50_ 1.3 - 2.0 Nilius et al. (2001); Hughes et al. (2018b)	Cryo-EM: 4.80 Å	6B5V Hughes et al. (2018b)	**S4:** L460
							**S6:** A561
	PCHPDs (cis-22a, Br-cis-22a, 3OG, 30, 31)	Human TRPV6	Inhibitors	IC_50_ 0.08 - 1.7 Simonin et al. (2015); Cunha et al. (2019); Bhardwaj et al. (2020); Cunha et al. (2020)	Cryo-EM: 3.10–4.34 Å	7K4B, 7K4C, 7K4D, 7K4E, and 7K4F Bhardwaj et al. (2020)	**S3:** P424, F425, and L428
							**S4:** F456, V459, C463, and M466
							**S4-S5:** T479, I482, Q483, and I486
							**S6:** M554, I557, T558, A561, and I565
	PCHPD (Br-cis-22a)	Rat TRPV6	Inhibitor	IC_50_ 0.96 ± 0.03 Bhardwaj et al. (2020)	X-ray: 3.70 Å	7D2K Bhardwaj et al. (2020)	**S3:** P424, F425, and L428
							**S4:** F456, V459, C463, and M466
							**S4-S5:** T479, I482, Q483, and I486
							**S6:** M554, I557, T558, A561, and I565
**(2)** DkTx site	DkTx	Rat TRPV1	Agonist	EC_50_ 0.14–0.24 Bohlen et al. (2010)	Cryo-EM: 2.95–3.84 Å	3J5Q, 5IRX, 7L2M, 7L2R, 7L2T, and 7L2U Cao et al. (2013); Gao et al. (2016); Zhang et al. (2021)	**P:** S629 and Y631
							**P-S6:** F649, N652, D654, and F655
							**S6:** K656, A657, V658, I660, and I661
**(3)** Extracellular vestibule recruitment sites	Ba^2+^	Rat TRPV6	Permeant ion	EC_50_ 1.91 ± 0.74 Saotome et al. (2016)	X-ray: 3.85 Å	5IWR Saotome et al. (2016)	**S1-S2:** D363
							**S5-P:** E518
							**P-S6:** D547
	Gd^3+^	Rat TRPV6	Channel blocker	IC_50_ 3.87 ± 0.83 Saotome et al. (2016)	X-ray: 3.80–3.90 Å	5IWT and 5WOA Saotome et al. (2016); Singh et al. (2017)	**S5-P:** D519 and E518
							**P-S6:** D547
**(4)** Extracellular pore entry site	NMDG	Rat TRPV1	Permeant ion	–	Cryo-EM: 3.64 Å	7L2V Zhang et al. (2021)	**P:** G645
	Ca^2+^	Rat TRPV6	Permeant ion	EC_50_ 1.47 ± 0.80 Saotome et al. (2016)	X-ray: 3.65–3.70 Å	5IWP and 5WO9 Saotome et al. (2016); Singh et al. (2017)	**P:** D541
	Ba^2+^	Rat TRPV6	Permeant ion	EC_50_ 1.91 ± 0.74 Saotome et al. (2016)	X-ray: 3.85 Å	5IWR Saotome et al. (2016)	**P:** D541
	Gd^3+^	Rat TRPV6	Channel blocker	IC_50_ 3.87 ± 0.83 Saotome et al. (2016)	X-ray: 3.80–3.90 Å	5IWT and 5WOA Saotome et al. (2016); Singh et al. (2017)	**P:** D541
**(5)** Selectivity filter site	NMDG	Rat TRPV1	Permeant ion	–	Cryo-EM: 3.26 Å	7L2X Zhang et al. (2021)	**P:** G643, M644, and G645
	Ca^2+^	Rat TRPV6	Permeant ion	EC_50_ 1.47 ± 0.80 Saotome et al. (2016)	X-ray: 3.65–3.70 Å	5IWP and 5WO9 Saotome et al. (2016); Singh et al. (2017)	**P:** T538
	Ba^2+^	Rat TRPV6	Permeant ion	EC_50_ 1.91 ± 0.74 Saotome et al. (2016)	X-ray: 3.85 Å	5IWR Saotome et al. (2016)	**P:** T538
	Ba^2+^	Frog TRPV4	Permeant ion	–	X-ray: 6.31 Å	6C8G Deng et al. (2018)	**P:** G675
	Cs^+^	Frog TRPV4	Permeant ion	–	X-ray: 6.50 Å	6C8F Deng et al. (2018)	**P:** G675
	Gd^3+^	Frog TRPV4	Channel blocker	–	X-ray: 6.50 Å	6C8H Deng et al. (2018)	**P:** G675
	Ruthenium red (RR)	Human TRPV6	Channel blocker	IC_50_ 9 ± 1 Hoenderop et al. (2001)	Cryo-EM: 2.43 Å	7S8B Neuberger et al. (2021a)	**P:** T539 and D542
**(6)** Central cavity site	Ca^2+^	Rat TRPV6	Permeant ion	EC_50_ 1.47 ± 0.80 Saotome et al. (2016)	X-ray: 3.65–3.70 Å	5IWP and 5WO9 Saotome et al. (2016); Singh et al. (2017)	**P:** T538
							**S6:** T566, M569, and L573
	Ba^2+^	Rat TRPV6	Permeant ion	EC_50_ 1.91 ± 0.74 Saotome et al. (2016)	X-ray: 3.85 Å	5IWR Saotome et al. (2016)	**P:** T538
							**S6:** T566, M569, and L573
**(7)** Intracellular pore entry site	PCHPDs (cis-22a, Br-cis-22a, 3OG, 30, 31)	Human TRPV6	Inhibitors	IC_50_ 0.08 - 1.7 Simonin et al. (2015); Cunha et al. (2019); Bhardwaj et al. (2020); Cunha et al. (2020)	Cryo-EM: 3.10–4.34 Å	7K4B, 7K4C, 7K4D, 7K4E, and 7K4F Bhardwaj et al. (2020)	**S6:** I575, A576, G579, and W583
	PCHPD (Br-cis-22a)	Rat TRPV6	Inhibitor	IC_50_ 0.96 ± 0.03 Bhardwaj et al. (2020)	X-ray: 3.70 Å	7D2K Bhardwaj et al. (2020)	**S6:** I575, A576, G579, and W583
	Calmodulin (CaM)	Rabbit TRPV5	Inactivator, channel blocker	–	Cryo-EM: 3.30–4.40 Å	6DMW and 6O20 Hughes et al. (2018a); Dang et al. (2019)	**S6:** W583
		Human TRPV6	Inactivator, channel blocker	–	Cryo-EM: 3.90 Å	6E2F Singh et al. (2018b)	**S6:** W583
		Rat TRPV6	Inactivator, channel blocker	–	Cryo-EM: 3.60 Å	6E2G Singh et al. (2018b)	**S6:** W582
**(8)** Deep S4-S5 site	Piperlongumine (PL)	Rat TRPV2	Inhibitor	IC_50_ 4.6 ± 0.13 Conde et al. (2021)	Cryo-EM: 3.46 Å	6WKN Conde et al. (2021)	**S4:** L513 and T516
							**S4-S5:** T522, Y525, S526, I529, and Q530
							**S5:** R539 and V543
							**S6:** V635, L636, and N639
**(9)** Shallow S4-S5 site	Econazole	Human TRPV6	Inhibitor	*IC*_50_ 4.39 ± 0.31 Neuberger et al. (2021a)	Cryo-EM: 2.85 Å	7S8C Neuberger et al. (2021a)	**S4:** V465, F468, and A469
							**S4-S5:** F472, M474, and L475
							**S5:** W495
**(10)** Deep portal site	Cannabidiol (CBD)	Rat TRPV2	Agonist	EC_50_ 3.7 Pumroy et al. (2019)	Cryo-EM: 3.20–3.40 Å	6U88 and 6U8A Pumroy et al. (2019)	**S5:** I533, L537, F540, L541, and Y544
							**P:** F601
							**S6:** L631, L632, Y634, V635, L637, L638, and M640
**(11)** Shallow portal site	ZINC17988990	Rabbit TRPV5	Inhibitor	*IC*_50_ 0.106 ± 0.027 Hughes et al. (2019)	Cryo-EM: 3.78 Å	6PBE Hughes et al. (2019)	**S4-S5:** F487, L490, and M491
							**S5:** C494 and W495
							**S6:** I565
	ZINC9155420	Rabbit TRPV5	Inhibitor	*IC*_50_ 2.91 ± 0.56 Hughes et al. (2019)	Cryo-EM: 4.20 Å	6PBF Hughes et al. (2019)	**S4-S5:** L490 and M491
							**S5:** C494
							**S6:** I564
**(12)** S1-S4 top site	2-APB	Mouse TRPV3	Agonist	EC_50_ 9–34 Singh et al. (2018a), Hu et al. (2009), Chung et al. (2004); Hu et al. (2004)	Cryo-EM: 4.24 Å	6DVZ Singh et al. (2018a)	**S1:** V458 and R462
							**S1-S2:** R464
							**S2:** R487
							**S3:** Y540
**(13)** S1-S4 base site	2-APB	Mouse TRPV3	Agonist	EC_50_ 9–34 Singh et al. (2018a), Hu et al. (2009), Chung et al. (2004); Hu et al. (2004)	Cryo-EM: 4.00–4.24 Å	6DVY and 6DVZ Singh et al. (2018a)	**S1:** S444
							**S2:** W493, K500, and E501
							**S3:** F526
							**S4:** Y565
		Human TRPV6	Inhibitor	*IC*_50_ 274 ± 27 Singh et al. (2018c)	Cryo-EM: 4.44 Å	6D7T Singh et al. (2018c)	**S2-S3:** Q418 and G422
							**S3:** G423 and H426
							**S4:** R470
							**TRP:** M603
		Rat TRPV6	Inhibitor	*IC*_50_ 184 ± 8 Singh et al. (2018c)	X-ray: 3.45–3.497 Å	6D7O and 6D7Q Singh et al. (2018c)	**S2:** E402
							**S3:** G422, F424, H425, and I428
							**S4:** N463, Y466, and R469
							**TRP:** M602
	2-APB-Br	Rat TRPV6	Inhibitor	–	X-ray: 3.60–4.30 Å	6D7V and 6D7X Singh et al. (2018c)	**S2:** E402
							**S3:** G422, F424, H425, and I428
							**S4:** N463, Y466, and R469
							**TRP:** M602
	Osthole	Mouse TRPV3	Competitive antagonist	IC_50_ 20–37 Sun et al. (2018); Neuberger et al. (2021b)	Cryo-EM: 3.64–3.99 Å	7RAS and 7RAU Neuberger et al. (2021b)	**S1:** S444
							**S2:** C496, I497, K500, and E501
							**S4:** Y565
							**TRP:** M706
	ZINC17988990	Rabbit TRPV5	Inhibitor	*IC*_50_ 0.106 ± 0.027 Hughes et al. (2019)	Cryo-EM: 3.78 Å	6PBE Hughes et al. (2019)	**LH1:** E294
							**S2:** L402, E403, and D406
							**S2-S3:** Y415 and L421
							**S3:** I429
							**S4:** Y467 and F468
							**TRP:** M603 and R606
**(14)** ARD-TMD linker site	2-APB	Mouse TRPV3	Agonist	EC_50_ 9–34 Singh et al. (2018a), Hu et al. (2009), Chung et al. (2004); Hu et al. (2004)	Cryo-EM: 4.00–4.24 Å	6DVY and 6DVZ Singh et al. (2018a)	**LH2:** H417, L420, and T421
							**Pre-S1:** H426 and H430
							**TRP:** R693 and L694
		Human TRPV3	Agonist	EC_50_ 28–78 Chung et al. (2004); Deering-Rice et al. (2014)	Cryo-EM: 3.60 Å	6OT5 Zubcevic et al. (2019b)	**LH2:** H417 and L420
							**Pre-S1:** H426 and L429
							**TRP:** R693
	Osthole	Mouse TRPV3	Competitive antagonist	IC_50_ 20–37 Sun et al. (2018); Neuberger et al. (2021b)	Cryo-EM: 3.64–3.99 Å	7RAS and 7RAU Neuberger et al. (2021b)	**LH2:** T421
							**Pre-S1:** H426, H430, and W433
							**TRP:** R693 and R696
**(15)** S2-S3 site	PIP_2_	Rabbit TRPV5	Agonist	–	Cryo-EM: 4.00 Å	6DMU Hughes et al. (2018a)	**ARD:** R302
							**S2-S3:** F416, G417, and Q418
							**S6:** R584
**(16)** ARD site	ATP	Rat TRPV1	Positive allosteric modulator	–	X-ray: 2.70–3.20 Å	2NYJ and 2PNN Lishko et al. (2007)	**ARD:** R115, F118, K155, K160, L163, N164, Y199, Q202, E210, and R211
		Human TRPV4	Positive allosteric modulator	–	X-ray: 2.95 Å	4DX2 Inada et al. (2012)	**ARD:** K192, K197, L200, N201, F231, Y236, Q239, and R248
	Desthiobiotin (DTB)	Rat TRPV6	Unknown	–	X-ray: 3.246–3.85 Å	5WO6, 5WO7, 5WO8, 5WO9, 5IWK, 5IWP, 5IWR, and 5IWT Saotome et al. (2016); Singh et al. (2017)	**ARD:** Q40, L88, M110, Y115, V151, and N158

### Vanilloid Site

**FIGURE 2:**
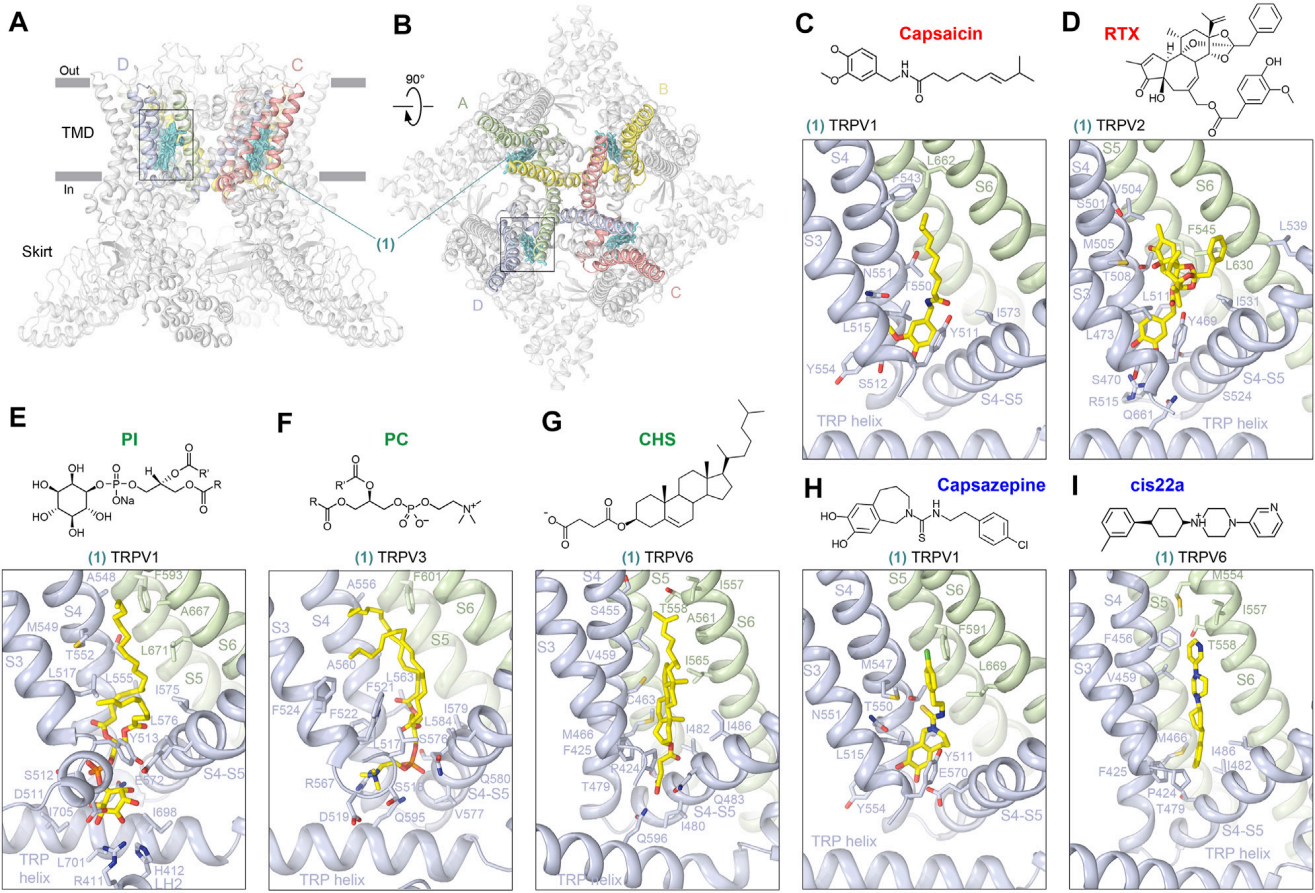
Vanilloid site. **(A–B)** Side **(A)** and top **(B)** views of the TRPV1 tetramer (gray; PDB ID: 7LQY) with the vanilloid site (1) ligands shown in cyan and domains contributing to this site (S3, S4, S4-S5, S5, and S6) shown in subunit colors (see Figures 1A,B). Rectangles indicate the region expanded in close-up views. **(C–I)** Close-up views of the vanilloid site in TRPV1 with bound capsaicin (**C**, PDB ID: 7LPA), in TRPV2 with bound RTX (**D**, PDB ID: 6BWJ), in TRPV1 with bound PI (**E**, PDB ID: 7LQY), in TRPV3 with bound PC (**F**, PDB ID: 7MIJ), in TRPV6 with bound CHS (**G**, PDB ID: 7S8B), in TRPV1 with bound capsazepine (**H**, PDB ID: 5IS0), and in TRPV6 with bound cis22a (**I**, PDB ID: 7K4B). Residues contributing to ligand binding are shown in sticks. The chemical structures of the corresponding ligands are shown above the close-up views.

The first ligand-binding site that has been identified structurally in TRP channels is the vanilloid site (Figure 2). This site (1) is located in the TMD region that faces the cytoplasmic leaflet of the membrane, in the crevice between S1-S4 and pore domains and is contributed mainly by S3, S4, S4-S5 linker, S5, and S6, often involving domains at the TMD–skirt interface, including LH2, S2-S3 loop, and TRP helix (Figures 2A, B; Table 1). The vanilloid site harbors agonists such as the active component of hot chili peppers, capsaicin, and its potent functional analog, resiniferatoxin (RTX), a naturally occurring chemical found in cactus-like resin spurge plants in Morocco and Northern Nigeria. Structures in complex with capsaicin were solved for rat (Cao et al., 2013; Kwon et al., 2021) and squirrel (Nadezhdin et al., 2021a) TRPV1 (Figure 2C, Supplementary Figure S1A). Interestingly, despite robust capsaicin-induced opening of TRPV1 in physiological experiments (Caterina et al., 1999; McIntyre et al., 2001; Premkumar et al., 2002; Savidge et al., 2002; Nadezhdin et al., 2021a), structures of TRPV1 bound to capsaicin alone have a non-conducting pore, unless another activating stimulus, heat, has been applied at the same time (Kwon et al., 2021). Even in this latter case, the pore is less open than in TRPV1 bound to two agonists simultaneously, RTX and double-knot toxin (DkTx) (Cao et al., 2013; Gao et al., 2016; Zhang et al., 2021). While certain conformational changes induced by capsaicin alone, including widening of the S6 bundle-crossing gate region (Cao et al., 2013) and π-to-α transition in S6 (Nadezhdin et al., 2021a), have been documented, the inability of capsaicin to open the channel completely suggests that as of now, preparations of TRPV1 protein for structural studies are not able to reproduce the natural environment of the plasma membrane or capture the channel action on the physiologically relevant time scale.

The situation with capsaicin is somewhat similar to the situation with RTX (Figure 2D, Supplementary Figure S1B), which is either ineffective or induces only partial opening of TRPV1 pore, being much less effective itself than in combination with DkTx (Cao et al., 2013; Gao et al., 2016; Zhang et al., 2021). The same is true for TRPV2, in which RTX induces conformational changes in the selectivity filter and gate region, often deviating from the four-fold rotational symmetry but has never produced the full opening of the ion channel pore (Zubcevic et al., 2018; Zubcevic et al., 2019a). The working hypothesis for the mechanism of vanilloid agonist action is that agonist binding induces movement of the S1-S4 bundle relative to the pore domain, which involves pulling the S4-S5 linker away from the central axis, altering the network of interactions between the P-loop, S5, and S6 and allowing the P-loop helices and S6 bundle crossing gate to widen the pore (Cao et al., 2013; Gao et al., 2016; Zubcevic et al., 2018; Zubcevic et al., 2019a; Zhang et al., 2021). This putative activation mechanism will likely become more precise and specific as one succeeds to identify structural conditions in which binding of a vanilloid agonist alone will lead to the channel pore opening.

In the absence of agonists (apo condition), the vanilloid-binding site is typically occupied by a lipid. In total, three types of lipids have been identified in the vanilloid site so far, and they appear to be TRPV subtype-specific. Indeed, in all apo-state TRPV1 structures with more or less clearly resolved density in the vanilloid site, the bound lipid was interpreted as phosphatidylinositol (PI) (Nadezhdin et al., 2021a; Kwon et al., 2021; Gao et al., 2016; Zhang et al., 2021) (Figure 2E, Supplementary Figure S1C). Since agonists have to expel PI from its binding site to activate TRPV1, PI might be considered an endogenous inhibitor of this channel. However, to test this hypothesis, one would have to activate TRPV1 in the absence of ligands by removing PI from the vanilloid site. More apparent inhibitory role can be assigned to the vanilloid site lipid in TRPV3, which resides in this site in the cold apo conditions but completely dissociates from this site upon activation by heat (Singh et al., 2019; Nadezhdin et al., 2021b). The vanilloid site lipid in TRPV3 has been modeled as phosphatidylcholine (PC) (Nadezhdin et al., 2021b; Shimada et al., 2020) (Figure 2F, Supplementary Figure S1D), but molecular dynamics (MD) simulations suggested rather low specificity of this site to different types of lipids (Nadezhdin et al., 2021b), which requires further studies. In higher resolution TRPV5-6 structures, the vanilloid site is occupied by densities of the characteristic shape that were modeled by either cholesterol or its soluble analog used in protein purifications, namely, cholesteryl hemisuccinate (CHS) (McGoldrick et al., 2018; Dang et al., 2019; Bhardwaj et al., 2020; Neuberger et al., 2021a) (Figure 2G, Supplementary Figure S2A). Whether the lipid at the vanilloid site plays a role of an agonist or inhibitor of TRPV5 and TRPV6 is currently unclear and will require additional functional experiments and structures at high resolution but in the absence of cholesterol/CHS.

The vanilloid site in TRPV channels not only binds agonists and lipids but also inhibitors, such as capsazepine in TRPV1 (Figure 2H, Supplementary Figure S2B) and cis22a in TRPV6 (Figure 2I, Supplementary Figure S2C). Capsazepine was proposed to act by displacing the resident PI lipid and stabilizing the interface between the S1-S4 and pore domains in the apo (closed) state-like conformation, which prevents pulling the S4-S5 linker away from the central axis to trigger conformational rearrangements that can lead to opening of the channel gate (Gao et al., 2016). For cis22a, as well as other representatives of (4-phenylcyclohexyl)piperazine derivatives (PCHPDs) that inhibit TRPV6-mediated currents with submicromolar affinity, the vanilloid site is modulatory, with the main inhibitory site located at the ion pore intracellular entrance (Bhardwaj et al., 2020). Because of the weak contribution of the vanilloid site to PCHPD inhibition, the mechanism of allosteric regulation through this site is not completely clear and requires additional studies. It has also been reported that the antifungal drug econazole binds to the vanilloid site in TRPV5 (Hughes et al., 2018b) (Supplementary Figure S2D). The corresponding 4.8-Å resolution structure allowed only an approximate placement of the compound into cryo-EM density (Hughes et al., 2018b), while more precise and confident fitting would require a higher resolution structure of the TRPV5–econazole complex. Such structure would be interesting to see, as the recent 2.85-Å resolution structure of the TRPV6–econazole complex has not identified the vanilloid site as a binding site for econazole (Neuberger et al., 2021a). Instead, the TRPV6 structure shows econazole binding to the shallow S4-S5 site, which is contributed by residues that are highly conserved between TRPV5 and TRPV6 channels (Neuberger et al., 2021a).

### Pore Sites

**FIGURE 3:**
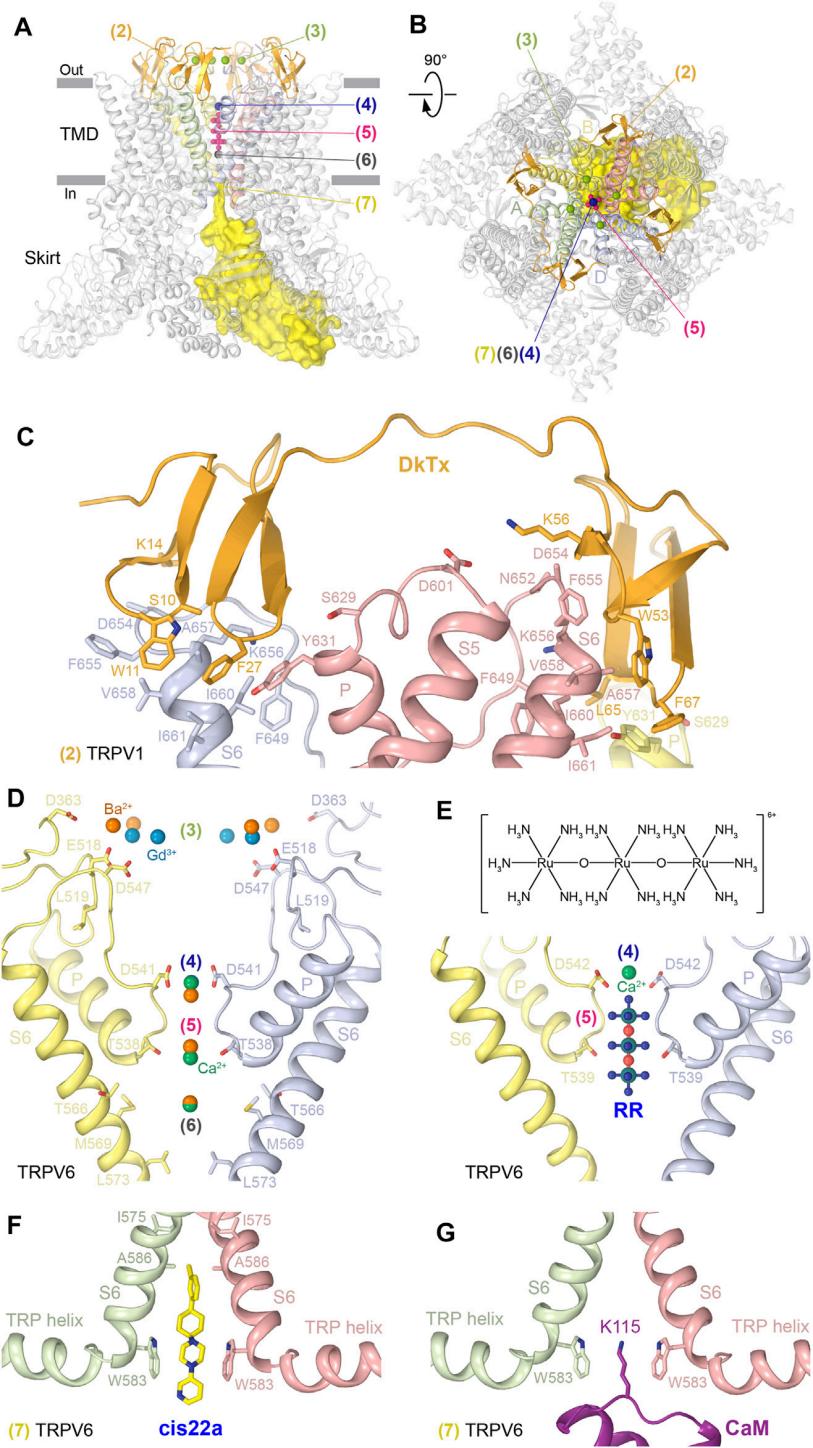
Pore sites. **(A–B)** Side **(A)** and top **(B)** views of the TRPV1 tetramer (gray; PDB ID: 7LQY) with the DkTx (2), extracellular vestibule recruitment (3), extracellular pore entry (4), selectivity filter (5), central cavity (6), and intracellular pore entry (7) sites illustrated by ligands in orange, green, blue, pink, gray, and yellow, respectively. **(C–G)** Close-up views of the DkTx site in TRPV1 with bound DkTx (**C**, PDB ID: 5IRX); extracellular vestibule recruitment, extracellular pore entry, selectivity filter, and central cavity sites in TRPV6 **(D)** with bound Ca^2+^ (PDB ID: 5WO9), Ba^2+^ (PDB ID: 5IWR), and Gd^3+^ (PDB ID: 5WOA); extracellular pore entry and selectivity filter sites in TRPV6 with bound Ca^2+^ and RR (**E**, PDB ID: 7S8B); and intracellular pore entry sites in TRPV6 with bound cis22a (**F**, PDB ID: 7K4B) or CaM (**G**, PDB ID: 6E2F). Residues contributing to ligand binding are shown in sticks. The chemical structure of RR is shown above the corresponding close-up view **(E)**.

The pore sites are contributed by the pore-forming P-loop and S6 as well as the extracellular loops (S1-S2, S5-P, and P-S6) that shape the ion channel extracellular vestibule (Figures 3A, B; Table 1). In TRPV1, two copies of the agonist DkTx bind to the extracellular side of the channel in a two-fold symmetrical manner, with each knot of this double-knot toxin binding to a site (2) composed of residues in the P-loop and S6 of two neighboring subunits (Cao et al., 2013; Gao et al., 2016; Zhang et al., 2021) (Figure 3C). The proposed mechanism of DkTx-induced TRPV1 opening suggests that DkTx binding induces a large backward tilting of the S1-S4 domain away from the channel central axis, which allosterically moves S5/S6 by pulling at the S4-S5 linker and opens the channel gate (Zhang et al., 2021). At the moment, it is unclear how DkTx interacts with the cap domain of TRPV1 and whether it affects permeation through the four portals leading to the ion conductance pathway (Nadezhdin et al., 2021a). Answering these questions will require structure determination of the full-length TRPV1 in complex with DkTx and with the completely resolved cap domain.

TRPV2-6 channels do not have a cap domain and instead have an extracellular vestibule leading to the pore entry. This vestibule contains binding sites for permeant cations, which in TRPV6 were resolved by X-ray crystallography (Saotome et al., 2016; Singh et al., 2017). Four symmetry-related cation binding sites or recruitment sites (3) were identified in TRPV6 as anomalous difference peaks for Ba^2+^ and Gd^3+^ (Figure 3D). These sites are contributed by negatively charged residues and have somewhat different locations, probably due to the difference in charge density. Although the anomalous difference peaks at the recruitment sites were not observed for Ca^2+^, presumably due to lower affinity, reduced occupancy, or weaker anomalous signal, the highly electronegative outer vestibule is likely involved in the general recruitment of cations toward the extracellular vestibule of the TRPV6 channel (Saotome et al., 2016). Consistent with the results of isothermal titration calorimetry experiments for Gd^3+^, the affinity of cations to the recruitment sites is lower than that to the binding sites in the pore (Saotome et al., 2016).

The next distinct binding site associated with the conductance pathway is the site at the pore extracellular entrance (4). In TRPV1, this site has been shown to bind a molecule of N-methyl-D-glucamine (NMDG), which appears as a blob of density in the cryo-EM map (Zhang et al., 2021). Strong blobs of density of a smaller size, interpreted as representing permeant cations, were observed at this location in the crystallographic and cryo-EM maps of TRPV5 (Hughes et al., 2018a; Dang et al., 2019; Hughes et al., 2018b; Hughes et al., 2019) and TRPV6 (Saotome et al., 2016; Singh et al., 2017; McGoldrick et al., 2018; Bhardwaj et al., 2020; Neuberger et al., 2021a; Singh et al., 2018b; Singh et al., 2018c). Solid evidence about the chemical identity of the pore entry site binders came from the crystallographic studies of TRPV6. Indeed, strong anomalous peaks at this location, contributed by side chains of the highly conserved aspartates (D542 in hTRPV6 and D541 in rTRPV6), were identified for the permeant cations Ca^2+^ and Ba^2+^ and for the channel blocker Gd^3+^ (Saotome et al., 2016), (Singh et al., 2017) (Figure 3D). The distance of 2.4 Å between Ca^2+^ and carboxylate oxygen of aspartate at this site matches the reported average Ca^2+^–oxygen distance in Ca^2+^-binding proteins (Yang et al., 2002), suggesting that the bound Ca^2+^ ion is at least partially dehydrated. Tight coordination of cations by the aspartates suggested a “knock-off” mechanism of ion permeation, strongly supported by MD simulations, which revealed 2 to 3 permeant ions continuously moving around the pore entry site to create the strong averaged density observed in crystallographic or cryo-EM maps (Sakipov et al., 2018). Because of the strong positive charge of Gd^3+^, its binding to the pore entry site is so tight that it cannot leave this site to permeate the channel (Sakipov et al., 2018).

Tight binding to the pore entry site not only explains the mechanism of TRPV6 block by Gd^3+^ but also why Gd^3+^ ions were not found 6–8 Å deeper in the pore, at the selectivity filter site (5), formed by backbone carbonyls and side chain hydroxyl groups of T538 in the lower parts of the P-loop extended region (Figure 3D). This selectivity filter site binds permeant cations Ca^2+^ and Ba^2+^, which were detected in the crystal structures of TRPV6 using anomalous difference Fourier maps (Saotome et al., 2016; Singh et al., 2017). The selectivity filter site in TRPV6 also binds the inorganic dye and low-specificity ion channel blocker ruthenium red (RR) (Neuberger et al., 2021a) (Figure 3E, Supplementary Figure S3A). RR occupies the entire selectivity filter, being surrounded by the carboxyl groups of D542 residues, the backbone carbonyl oxygens of I541, I540, and T539 residues, and the hydroxyl groups of T539 residues, which all together create a highly electronegative environment favorable for the positively charged RR that carries a 6^+^ total charge. RR not only plugs the pore to prevent ion conductance, but it also causes the closure of the lower gate, with the characteristic π-to-α transition in S6 that typically accompanies channel closure (McGoldrick et al., 2018; Neuberger et al., 2021a). A possible explanation of why this happens is that RR creates an electric field inside the pore’s central cavity that interacts with the electric dipole of the S6 helix. This interaction causes a repulsion of the lower portion of S6 away from RR, which results in rotation of the lower portion of S6 and helps S6 to become entirely α-helical (Neuberger et al., 2021a).

Another binding site in the TRPV6 pore (6) is 6–7 Å below the selectivity filter site, in the middle of the central cavity (Figure 3D). The corresponding anomalous difference peaks were found for both Ca^2+^ and Ba^2+^, although the anomalous peak for Ca^2+^ was less robust, presumably due to its weaker anomalous diffraction properties. The anomalous signal at the central cavity site also suggests that cations bound here are ordered by water molecules, which can be held in place by weak hydrogen-bonding interactions and pore helix dipoles, pointing their partial negative charges toward the middle of the central cavity, similar to K^+^ channels (Doyle et al., 1998).

There is also a site at the pore intracellular entrance (7), which is formed by the S6 helices and represents the main site of TRPV6 inhibition by PCHPDs (Bhardwaj et al., 2020) (Figure 3F, Supplementary Figure S3B). When bound to this site, PCHPDs plug the TRPV6 pore and prevent ion conductance. In addition, interactions of the positive charge of PCHPDs with the π orbitals of tryptophan (W583 in hTRPV6 and W582 in rTRPV6) indole rings, which form a cubic cage at the intracellular pore entrance, pull the S6 helices toward the pore center. This pore narrowing brings isoleucines (I575 in hTRPV6 and I574 in rTRPV6) close to each other to hydrophobically seal the channel, mimicking the pore conformation of the TRPV6-inactivated state. Indeed, when calcium-binding protein calmodulin (CaM) binds at the intracellular pore entrance to inactivate the channel, it inserts the side chain of its lysine K115 into the center of the cubic tryptophan cage (Singh et al., 2018b). The positively charged ε-amino group of K115 makes an atypically strong cation–π interaction with the π-system of four tryptophan indole rings (Figure 3G). TRPV5 has a mechanism of inactivation by CaM similar to TRPV6 (Hughes et al., 2018a; Dang et al., 2019), while structures of other TRPVs in complex with CaM have not been solved, and it remains to be discovered whether they have a similar mechanism of inactivation.

### S4-S5 Sites

**FIGURE 4:**
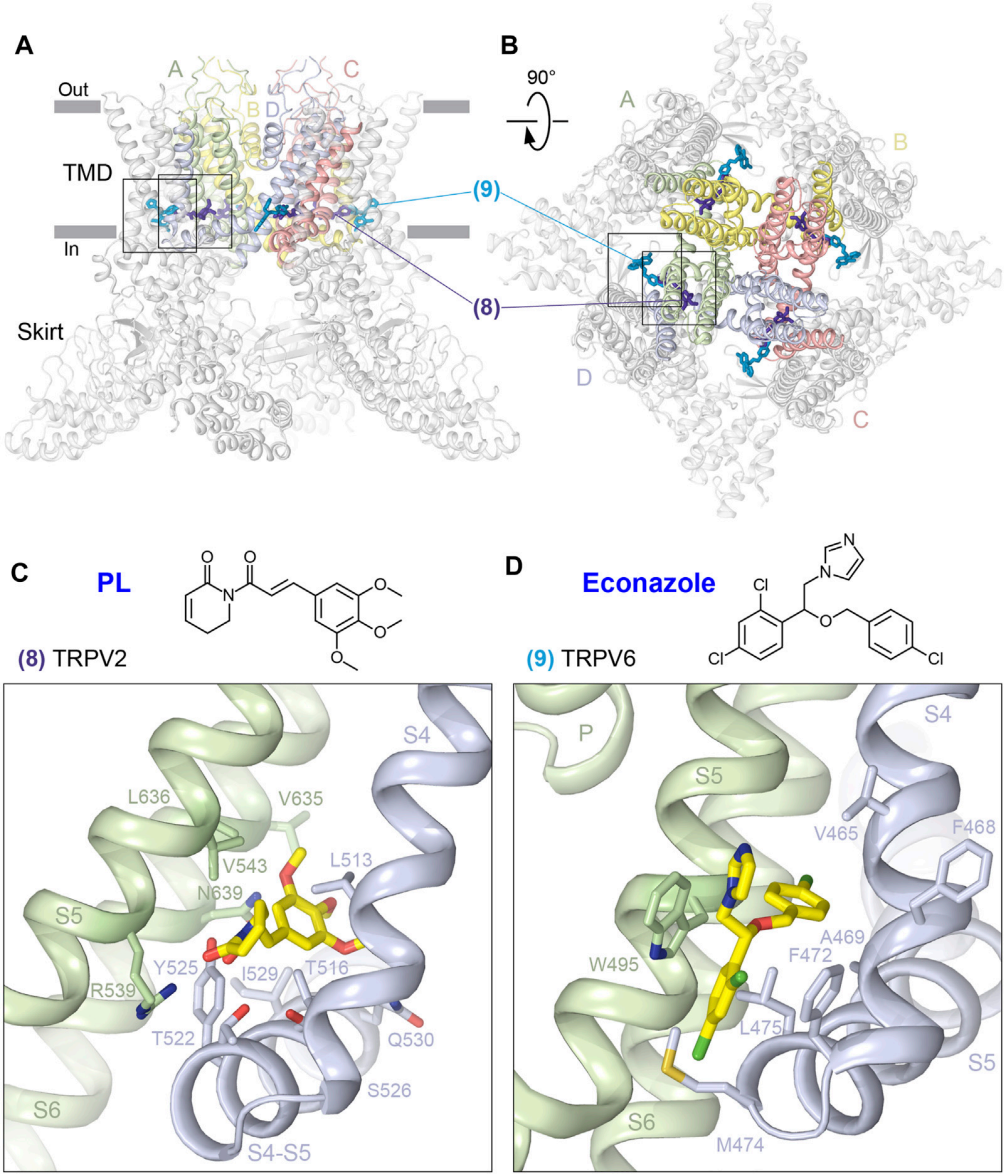
S4-S5 sites. **(A–B)** Side **(A)** and top **(B)** views of the TRPV1 tetramer (gray; PDB ID: 7LQY) with the S4-S5 deep (8) and shallow (9) sites illustrated by ligands in dark and light blue, respectively. Rectangles indicate the regions expanded in close-up views. **(C-D)** Close-up views of the deep S4-S5 site in TRPV2 with bound piperlongumine **(C)** (PDB ID: 6WKN) and shallow S4-S5 site in TRPV6 with bound econazole **(D)** (PDB ID: 7S8C). Residues contributing to ligand binding are shown in sticks. The chemical structures of the ligands are shown above the close-up views.

Two types of sites are located right above the S4-S5 linker, where ligands bind at the interface between S4 and S5 of two neighboring subunits (Figures 4A,B; Table 1). At one site (8), the ligand wedges deeper into the interface and reaches residues on S6. An example of such ligand is the alkaloid piperlongumine (PL), which produces potent allosteric inhibition of TRPV2, presumably underlying the anticancer activity of PL in mouse models of glioblastoma (Conde et al., 2021). PL makes contacts with residues in S4 and S4-S5 linker of one TRPV2 subunit and S5 and S6 of the neighboring subunit (Figure 4C, Supplementary Figure S4A). By wedging itself between S4 and S5 of the neighboring subunits, PL pushes the S4-S5 linker down, shifting it by ∼4 Å toward S5 and causing a downward movement of the entire intracellular skirt by ∼3 Å. According to the proposed mechanism of PL action, PL inhibits TRPV2 by immobilizing the S4-S5 linker and locking the channel in a desensitized state (Conde et al., 2021).

At the second, shallower site (9), ligands do not wedge deeply into the interface between S4 and S5 of two neighboring subunits and correspondingly do not reach S6. An example is the binding site of the antifungal drug econazole, which produces strong inhibition of TRPV6-mediated currents (Nilius et al., 2001) (Figure 4D, Supplementary Figure S4B). By binding to the shallow S4-S5 site in TRPV6, where it is sandwiched between W495 of S5 and F472 of S4 and surrounded by hydrophobic side chains of L496 and V499 of S5 as well as M466, A469, M474, and L475 of S4, econazole replaces a lipid that is otherwise present at this location (Neuberger et al., 2021a). Because of the smaller size of econazole than the lipid, econazole binding creates a void that allows Q473 and M474 side chains to move closer to D472 and W495 and correspondingly away from R589. Separation of Q473 and R589 results in breakage of the salt bridge connecting these residues in the open state (McGoldrick et al., 2018). The loss of this salt bridge, which energetically compensates for the unfavorable α-to-π transition in S6, reverses the transition. Concurrently, the lower parts of S6 helices rotate by ∼100°, leading to the separation of D489 and T581 and the loss of the open-state-stabilizing hydrogen bond between them (McGoldrick et al., 2018). The rotated lower parts of S6 expose the side chains of L574 and M578 toward the pore center, and they hydrophobically seal the pore to prevent ion conductance, thus converting the channel into the closed, non-conducting state (Neuberger et al., 2021a).

### Portal Sites

**FIGURE 5:**
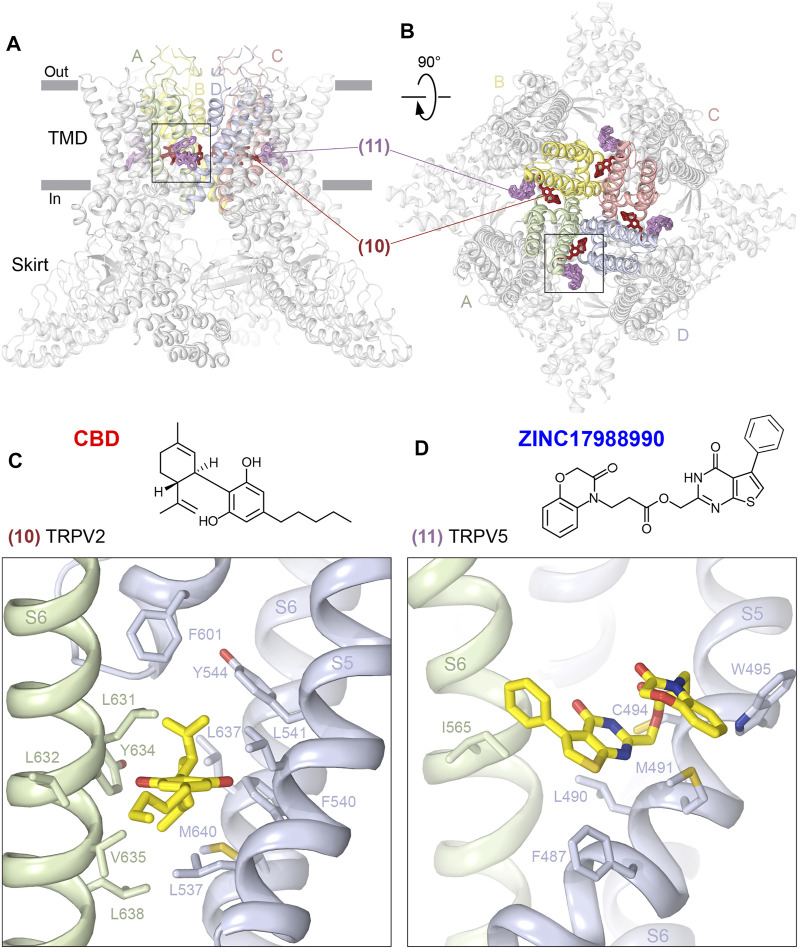
Portal sites. **(A–B)**, Side **(A)** and top **(B)** views of the TRPV1 tetramer (gray; PDB ID: 7LQY) with the deep (10) and shallow (11) portal sites illustrated by ligands in brown and pink, respectively. Rectangles indicate the region expanded in close-up views. **(C–D)**, Close-up views of the deep portal site in TRPV2 with bound cannabidiol (**C**, PDB ID: 6U8A) and the shallow portal site in TRPV5 with bound ZINC17988990 (**D**, PDB ID: 6PBE). Residues contributing to ligand binding are shown in sticks. The chemical structures of the ligands are shown above the close-up views.

There are two types of sites that bind to deep or shallow parts of the portals that connect the membrane environment surrounding the channel to the central cavity of the channel pore (Figures 5A,B; Table 1). The deep portal site (10) is contributed by S6 of one subunit and S5, P-loop and S6 of the neighboring subunit, and in TRPV2 is occupied by the agonist cannabidiol (CBD), a natural product of the *Cannabis sativa* plant (Pumroy et al., 2019). The CBD site in TRPV2 is lined with mostly hydrophobic and aromatic residues, including L631, L632, Y634, V635, and L638 from S6 of one protomer and L537, F540, L541, and Y544 from S5, L637 and M640 from S6, and F601 from the P-loop of another protomer (Figure 5C, Supplementary Figure S5A). While CBD-bound TRPV2 structures showed structural differences compared to apo structures, they nevertheless had a non-conducting pore and the corresponding channel conformations were interpreted as pre-open or desensitized states (Pumroy et al., 2019). Accordingly, understanding of the mechanism of TRPV2 activation by CBD awaits additional structural studies.

The shallow portal site (11) is contributed by S6 of one subunit and S4-S5 linker and S5 of the neighboring subunit, and in TRPV5 it is occupied by synthetic inhibitors ZINC9155420 and ZINC17988990 identified through structure-based virtual screening (Hughes et al., 2019). Similar to the deep portal site, the shallow portal site in TRPV5 is lined with mostly hydrophobic and aromatic residues, which for ZINC17988990 include I565 from S6 of one protomer, F487, L490, and M491 from S4-S5 linker, and C494 and W495 from S5 of another protomer (Figure 5D, Supplementary Figure S5B). Mutation of M491 to alanine produced only a small decrease in the inhibitory potency of ZINC17988990, suggesting that the shallow portal site is unlikely to play a major role in ZINC17988990-mediated inhibition of TRPV5, which instead is accomplished through binding to another site at the base of the S1-S4 bundle (Hughes et al., 2019) (see the following).

### S1-S4 Sites

**FIGURE 6:**
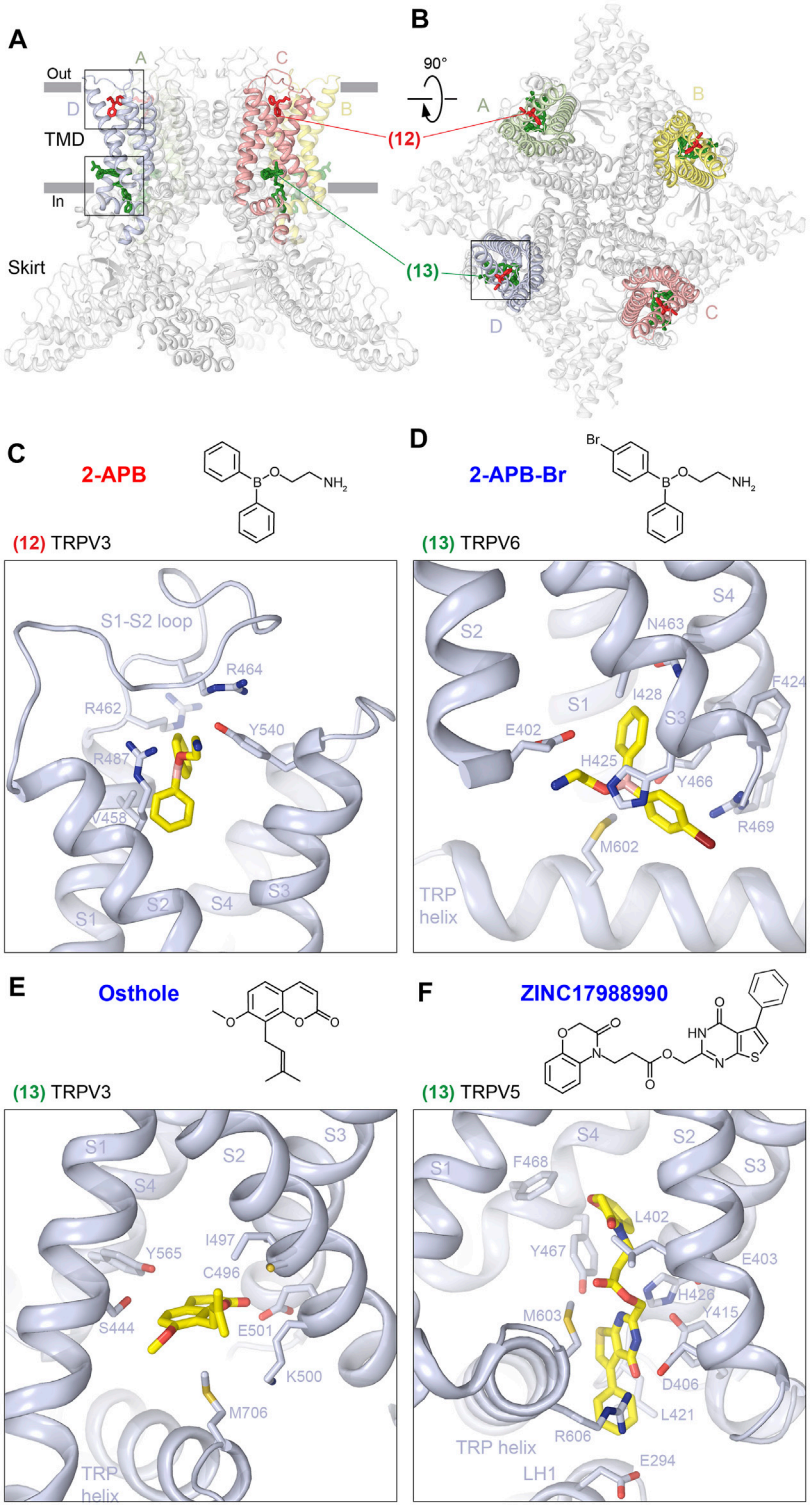
S1-S4 sites. **(A–B)**, Side **(A)** and top **(B)** views of the TRPV1 tetramer (gray; PDB ID: 7LQY) with the S1-S4 top (12) and base (13) sites illustrated by ligands in red and green, respectively. Rectangles indicate the regions expanded in close-up views. **(C–F)** Close-up views of the S1-S4 top site in TRPV3 with bound 2-APB (**C**, PDB ID: 6DVZ) and S1-S4 base site in TRPV6 with bound 2-APB-Br (**D**, PDB ID: 6D7V), in TRPV3 with bound osthole (**E**, PDB ID: 7RAS), and in TRPV5 with bound ZINC17988990 (**F**, PDB ID: 6PBE). Residues contributing to ligand binding are shown in sticks. The chemical structures of the corresponding ligands are shown above the close-up views.

The top and base of the S1-S4 domain represent two locations at the membrane–solute interface convenient for binding different chemical nature compounds to regulate the function of TRPV channels (Figures 6A,B; Table 1). The top S1-S4 site (12) has been shown to be important for the activation of mouse TRPV3 by 2-APB (Singh et al., 2018a). 2-APB binds at this site by substituting the S1-S2 loop, which resides in the S1-S4 top pocket in the apo state. 2-APB binding involves residues V458 and R462 from S1, R464 from the S1-S2 loop, R487 from S2, and Y540 from S3 (Figure 6C, Supplementary Figure S6A). Mutations of R487 and Y540 as well as of the close-proximity residue Q483 to alanine produced strong reduction in potency of 2-APB activation, supporting an important role of this site in mouse TRPV3 activation (Singh et al., 2018a). According to the proposed mechanism of TRPV3 activation, 2-APB outcompetes the S1-S2 loop and wedges into and expands the top of the S1-S4 bundle. As the top of the S1-S4 bundle expands, the cleft between S4 and the neighboring S6 narrows, squeezing out a lipid from the vanilloid site, and the S1-S4 and pore domain interface rearranges in a manner that supports channel opening (Singh et al., 2018a). Interestingly, 2-APB binding to the top S1-S4 site was not observed in human TRPV3, where it was only identified bound to one of the other two 2-APB binding sites identified in mouse TRPV3, the ARD-TMD site (see below).

The second binding site of agonist 2-APB in mouse TRPV3 is at the base of the S1-S4 bundle (13), which is contributed by residues in S1, S2, S3, and S4 (Table 1). Binding of 2-APB to this site in mouse TRPV3 does not introduce strong conformational changes but is likely necessary for the relative stability of the pore, S1-S4 and skirt domains during channel opening. Supporting the important role of this site in activation of mouse TRPV3, the Y564A mutation produces a strong increase in 2-APB potency (Singh et al., 2018a). In TRPV6, 2-APB and its brominated analog, 2-APB-Br, bind to the base S1-S4 site to produce inhibition of this ion channel. Along with contribution of residues from S2 (E402), S3 (G422, F424, H425, and I428), and S4 (N463, Y466, and R469), like in TRPV3, the inhibitor binding also involves the residue M602 from the TRP helix (Figure 6D, Supplementary Figure S6B). Similar to the activation of TRPV3, the mechanism of TRPV6 inhibition by 2-APB assumes an active role of lipids. Presumably, 2-APB binding displaces a lipid that resides at this site, thus promoting the formation of a hydrophobic cluster contributed by residues from S3, S4, and the S4-S5 linker. The formation of the cluster displaces the activating lipid from the vanilloid site and eliminates hydrogen bonds, which stabilize the open state by energetically compensating the unfavorable α-to-π transition in S6. As S6 turns α-helical, the channel closes and its pore becomes impermeable to ions (Singh et al., 2018c).

Interestingly, the base S1-S4 site in TRPV3 harbors not only the agonist 2-APB but also the inhibitor osthole, which thus can be considered as a competitive antagonist (Figure 6E, Supplementary Figure S6C). The coumarin osthole is an active ingredient of *Cnidium monnieri* as well as other medicinal plants, which shows antipruritic and anticancer activity and is used in traditional Chinese medicine for the treatment of skin-related diseases (You et al., 2009; Zhang et al., 2015; Yang et al., 2016; Shokoohinia et al., 2018; Sun et al., 2018). Osthole binding at the base of S1-S4 involves S444 from S1, C496, I497, K500, and E501 from S2, Y565 from S4, and M706 from the TRP helix, and produces separations of S1 and S2 as well as pre-S1 and the TRP helix (Neuberger et al., 2021b). These separations lead to movement of S3 and S4 away from the pore center, followed by movement of S5 and S6, which in turn has two consequences. First, it results in a dramatic widening of the upper pore, including the selectivity filter. Second, S5 and S6 dislocation leads to movement of the TRP helix and a kink in the S4-S5 linker, which allows to disconnect the upper pore from the gate region. A concomitant increase in separation of S5 and S6 is accompanied by a π-to-α transition in S6 and rotation of its lower portion by ∼100°, with M677 side chains now facing the pore center, hydrophobically sealing it for ion conduction and finalizing the open to closed state conversion. During all these changes, the structural environment of the base S1-S4 site when it binds to 2-APB and osthole appears to be similar (Singh et al., 2018a; Neuberger et al., 2021b), suggesting that this site is modulatory and unlikely to cause drastic structural rearrangements. In contrast, the top S1-S4 site appears to be structurally different in 2-APB- and osthole-bound structures and likely plays the most critical role in the osthole-induced TRPV3 inhibition, even though osthole density at this site was not resolved. More work is required to better understand the mechanism of TRPV3 inhibition by osthole.

In TRPV5, the base S1-S4 site was proposed to bind a synthetic inhibitor, ZINC17988990 (Hughes et al., 2019). Because of the larger size of ZINC17988990, its binding pocket is larger than the one for 2-APB, 2-APB-Br, and osthole (Figures 6D, E), extends intracellularly, and along with the residues from S2 (L402, E403, and D406), S2-S3 loop (Y415 and L421), S3 (I429), S4 (Y467 and F468), and TRP helix (M603 and R606) involves E294 from LH1 (Figure 6F, Supplementary Figure S6D). In contrast to the shallow portal site, which appears to play a minor role in TRPV5 inhibition by ZINC17988990, the base S1-S4 site was proposed to serve as the primary binding pocket through which ZINC17988990 exerts its inhibitory effect (Hughes et al., 2019). Indeed, mutations D406A and Y415F resulted in reduced potency of ZINC17988990 inhibition (Hughes et al., 2019). According to the proposed mechanism of TRPV5 inhibition by ZINC17988990, positioning of the inhibitor at the base S1-S4 site limits the activation-associated conformational changes in the channel induced by PI(4,5)P_2_, including movement of the S1-S4 domain away from the pore axis, with the largest translation observed for the lower halves of the S1 and S2 helices, a counterclockwise rotation of the bundle, and a pivot of the TRP helix (Hughes et al., 2018a). Consequently, the antagonist plays the role of a molecule that locks the channel in the inhibited state, which is inconsistent with the putative activation-associated conformational changes (Hughes et al., 2019).

### Ankyrin Repeat Domain–Transmembrane Domain Interface Sites

**FIGURE 7:**
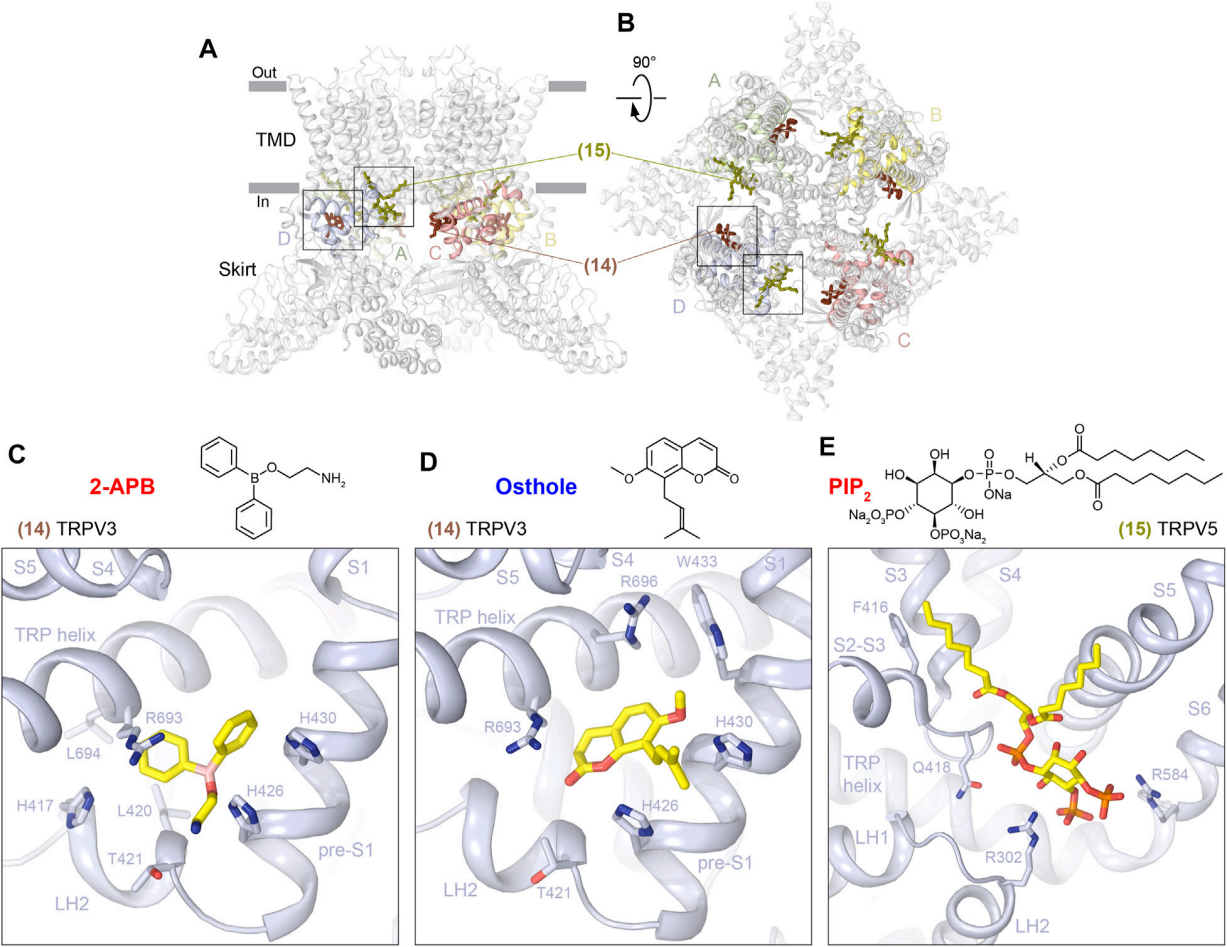
ARD–TMD interface sites. **(A–B)** Side **(A)** and top **(B)** views of the TRPV1 tetramer (gray; PDB ID: 7LQY) with the ARD-TMD linker (14) and S2-S3 (15) sites illustrated by ligands in brown and olive, respectively. Rectangles indicate the regions expanded in close-up views. **(C–E)** Close-up views of the ARD-TMD linker site in TRPV3 with bound 2-APB (**C**, PDB ID: 6DVZ) or osthole (**D**, PDB ID: 7RAS) and the S2-S3 site in TRPV5 with bound PIP_2_ (**E**, PDB ID: 6DMU). Residues contributing to ligand binding are shown in sticks. The chemical structures of the corresponding ligands are shown above the close-up views.

The ARD–TMD interface sites (14 and 15) are located more intracellularly than all the sites described previously (Figure 1), but they remain in close proximity to the intracellular membrane border and are contributed by the closest to the TMD regions of the ARD, the LH2, and pre-S1 elements of the ARD-TMD linker domain, the S2-S3 linker, and the TRP helix (Figures 7A, B; Table 1). In TRPV3, the ARD–TMD interface site (14) is located at the nexus of the linker domain, contributing LH2 and pre-S1, and TRP helix, and binds to the agonist 2-APB (Figure 7C, Supplementary Figure S7A) and antagonist osthole (Figure 7D, Supplementary Figure S7B). This site was first identified through mutagenesis (Hu et al., 2009), and in mouse TRPV3 appears to play a modulatory role (Singh et al., 2018a; Neuberger et al., 2021b). In contrast, the ARD-TMD linker site is the only site in human TRPV3 that binds 2-APB and was correspondingly proposed to drive activation-associated conformational changes (Zubcevic et al., 2019b). According to the putative mechanism of human TRPV3 activation by 2-APB, binding of the agonist between pre-S1 and TRP helix increases coupling between the linker domain and the TRP helix. The increased coupling is associated with a swivel of the TRP helix relative to S6, which causes S6 movement and conversion of the closed state to the open state (Zubcevic et al., 2019b).

The second representative of the ARD-TMD interface sites is the S2-S3 site (15) that in TRPV5 was proposed to bind PI(4,5)P_2_ (Hughes et al., 2018a) (Figure 7E, Supplementary Figure S7C). Apart from the S2-S3 linker itself (F416, G417, and Q418), this PI(4,5)P_2_ binding site is contributed by the ARD (R302) and S6 (R584). Binding of PI(4,5)P_2_ to the S2-S3 site was supported by MD simulations and mutagenesis (Hughes et al., 2018a). Comparing the PI(4,5)P_2_-bound and apo state structures, it was proposed that PI(4,5)P_2_ uses its phosphate group to form a salt bridge with R584 and pulls S6 away from the center of the pore. According to the proposed mechanism, the resulting extension and rotation of S6 pulls W583 out of the pore and stabilizes its position through interaction with Q587, thus opening the lower gate (Hughes et al., 2018a).

### Ankyrin Repeat Domain Site

**FIGURE 8:**
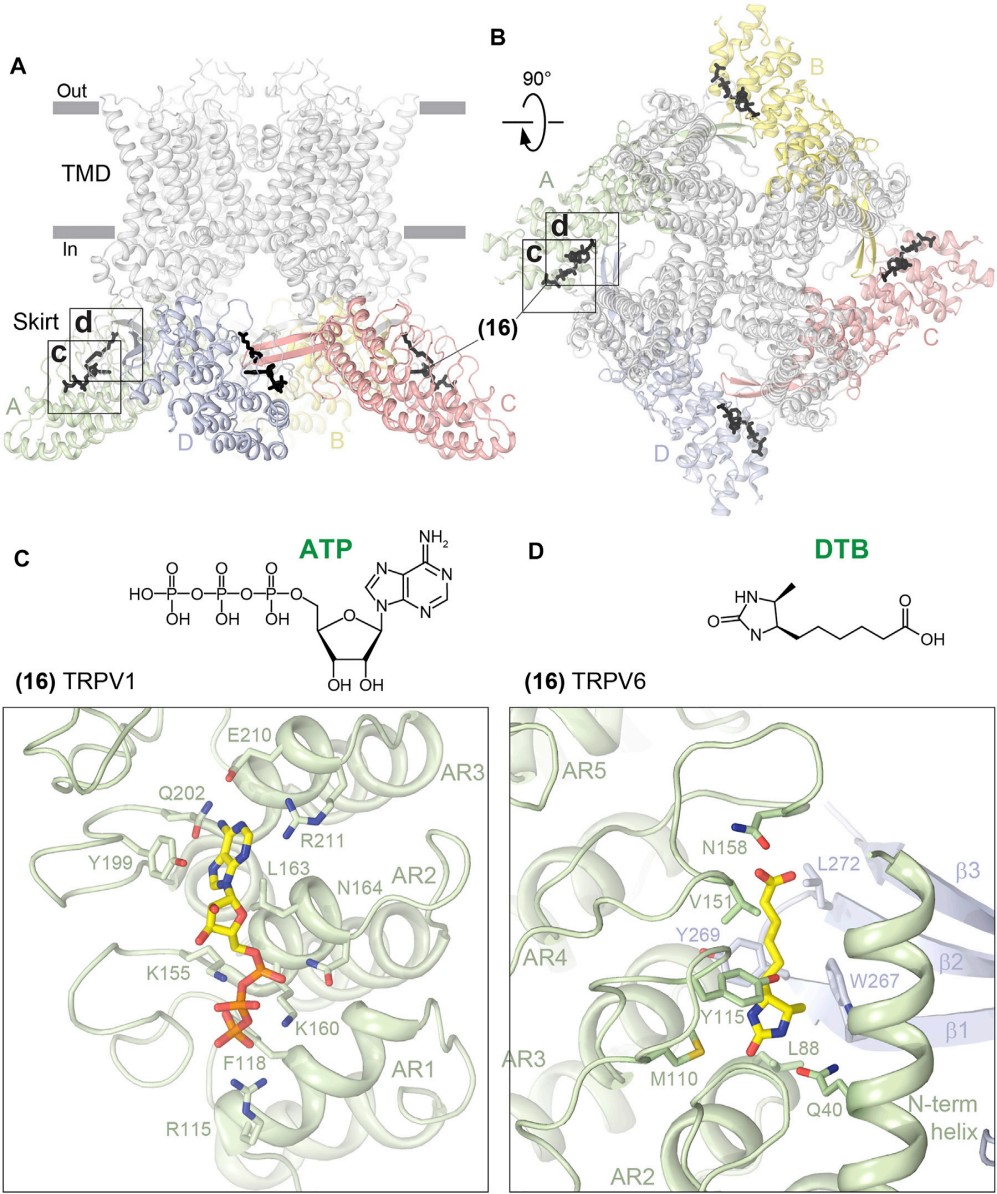
ARD site. Side **(A)** and top **(B)** views of the TRPV1 tetramer (gray; PDB ID: 7LQY) with the ARD site (16) illustrated by ligands in black. Rectangles indicate the regions expanded in close-up views. **(C–D)** Close-up views of the ARD site in TRPV1 with bound ATP (**C**, PDB ID: 2PNN) and in TRPV6 with bound desthiobiotin (**D**, PDB ID: 5WO9). Residues contributing to ligand binding are shown in sticks. The chemical structures of the ligands are shown above the close-up views.

Among all the sites discovered in TRPV channels, the only one that is distal from the TMD is the ARD site (16). Correspondingly, this site is contributed by the ARD residues only (Figures 8A, B; Table 1). Using crystal structures of the isolated ARD, the ARD site was shown to bind ATP at similar locations in TRPV1 (Lishko et al., 2007) (Figure 8C, Supplementary Figure S8A) and TRPV4 (Inada et al., 2012). When mapped on the full-length channels, this site is somewhat close to the three-stranded β-sheet but makes no direct contact with its residues and is fairly distal from the channel gate. It was hypothesized that ATP competes for binding to the ARD site with CaM, which causes desensitization of TRPV1 (Lishko et al., 2007). Unfortunately, no structures of CaM- or ATP-bound full-length TRPV1 or 4 are available, making it difficult to speculate about the possible structural mechanism of channel regulation by ATP.

The ARD site in TRPV6 is similar to the ATP-binding sites in TRPV1 and 4 and has been proposed to bind desthiobiotin (DTB) (Saotome et al., 2016; Singh et al., 2017) (Figure 8D, Supplementary Figure S8B). DTB was included as an eluent in the rat TRPV6 affinity purification procedure and has not been shown to play physiologically relevant roles. It is possible that the same site can be occupied by endogenous molecules such as ATP or synthetic molecules of a similar size to DTB. TRPV function may be affected by such binding through altering the intersubunit contacts mediated by the ARD and the three-stranded β-sheet. Future studies are required to explore the potential of the ARD site for TRPV regulation for scientific and medicinal purposes.

### Outlook

We described 16 unique ligand-binding sites that have been structurally identified in TRPV channels using X-ray crystallography and cryo-EM. Many of these sites have been discovered very recently, and we look forward to seeing more examples of ligands that bind to these sites and perhaps the discovery of new sites in the near future. With the progress in genome sequencing, it became obvious that some of these sites can change due to altered amino-acid composition as a result of single nucleotide polymorphisms (SNPs) and disease mutations. Many of these changes result in altered trafficking, expression, and gain or loss of channel function and include mutations in TRPV3 causing Olmsted syndrome, mutations in TRPV4 leading to skeletal diseases, such as dysplasias, and neuropathies, such as Charcot–Marie–Tooth disease type 2C, and mutations in TRPV6 associated with pancreatitis and skeletal abnormalities as well as SNPs in TRPV5 and TRPV6 causing altered calcium transport (Na et al., 2009; Cantero-Recasens et al., 2010; Loh et al., 2013; Nilius and Voets, 2013; Na and Peng, 2014; Loukin et al., 2015; Sadofsky et al., 2017; Wang and Wang, 2017; Nett et al., 2021; Zhong et al., 2021). These changes in the ligand-binding sites have to be taken into account when designing new ligands to target specific disease conditions or SNPs.

It has also become clear that due to similarity in structural architecture, especially in the TMD, many of the 16 unique sites identified in TRPV channels are also present in other subfamilies of TRP channels and bind ligands, which are often different and specific to those subfamilies or their select members. For the vanilloid site, examples include TRPA1 agonist GNE551 (Liu et al., 2021) and TRPM5 antagonist NDNA. A homologous to the DkTx site in TRPC6 binds the agonist AM-0883 (Bai et al., 2020). The deep S4-S5 site in TRPC6 harbors the inhibitor BTDM (Guo et al., 2022). The deep portal site binds the agonist ML-SA1 (Schmiege et al., 2017; Zhou et al., 2017) and inhibitor ML-SI3 (Schmiege et al., 2021) in TRPML1, inhibitors HC-070 (Song et al., 2021) and Pico145 (Wright et al., 2020) in TRPC5, and A-967079 (Paulsen et al., 2015) in TRPA1. Many ligands have been found to bind to the S1-S4 base site, including Ca^2+^ that plays a role of an activator in TRPC3, TRPM2, and TRPM4 and an inhibitor in TRPM5 (Autzen et al., 2018; Huang et al., 2018; Wang et al., 2018; Zhang et al., 2018; Ruan et al., 2021; Guo et al., 2022), Na^+^ in TRPC4 (Duan et al., 2018), a cation in TRPC5 (Duan et al., 2019), the inhibitors clemizole in TRPC5 (Song et al., 2021), and SAR7334 (Guo et al., 2022) and AM-1473 (Bai et al., 2020) in TRPC6 as well as the agonists WS-12 and icilin (Yin et al., 2019) and inhibitors TC-I 2014 and AMTB (Diver et al., 2019) in TRPM8. Therefore, the described 16 sites can be used as templates for the structure-based drug design not only in TRPV channels but also in all representatives of the TRP channel family. Rapid development of structural pharmacology, combined with recent advances in computation, structure prediction, artificial intelligence, and machine-learning methods, will foster the discovery of new pharmacological agents to combat human diseases.

## Supplementary Material

Supplementary Figure S1 LigPlot^+^ analysis for the vanilloid site (part 1). LigPlot^+^ diagrams for capsaicin bound to TRPV1 **(A)** (PDB ID: 7LPA), RTX bound to TRPV2 **(B)** (PDB ID: 6BWJ), PI bound to TRPV1 **(C)** (PDB ID: 7LQY), and PC bound to TRPV3 **(D)** (PDB ID: 7MIJ). Residues that belong to a specific structural domain are grouped in highlighted islands (green) and labeled. The analysis was performed using LigPlot^+^ software (https://www.ebi.ac.uk/thornton-srv/software/LigPlus/).

Supplementary Figure S2 LigPlot^+^ analysis for the vanilloid site (part 2). LigPlot^+^ diagrams for CHS bound to TRPV6 **(A)** (PDB ID: 7S8B), capsazepin bound to TRPV1 **(B)** (PDB ID: 5IS0), cis22a bound to TRPV6 **(C)** (PDB ID: 7K4B), and econazole bound to TRPV5 **(D)** (PDB ID: 6B5V). Residues that belong to a specific structural domain are grouped in highlighted islands (green) and labeled. The analysis was performed using LigPlot^+^ software (https://www.ebi.ac.uk/thornton-srv/software/LigPlus/).

Supplementary Figure S3 LigPlot^+^ analysis for the pore site. LigPlot^+^ diagrams for RR bound to the selectivity filter site **(A)** (PDB ID: 7S8B) and cis22a bound to the intracellular pore entry site **(B)** (PDB ID: 7K4B) in TRPV6. Residues that belong to a specific structural domain are grouped in highlighted islands (green) and labeled. The analysis was performed using LigPlot^+^ software (https://www.ebi.ac.uk/thornton-srv/software/LigPlus/).

Supplementary Figure S4 LigPlot^+^ analysis for the S4-S5 sites. LigPlot^+^ diagrams for piperlongumine bound to the deep S4-S5 site in TRPV2 **(A)** (PDB ID: 6WKN) and econazole bound to the shallow S4-S5 site in TRPV6 **(B)** (PDB ID: 7S8C). Residues that belong to a specific structural domain are grouped in highlighted islands (green) and labeled. The analysis was performed using LigPlot^+^ software (https://www.ebi.ac.uk/thornton-srv/software/LigPlus/).

Supplementary Figure S5 LigPlot^+^ analysis for the portal sites. LigPlot^+^ diagrams for cannabidiol bound to the deep portal site in TRPV2 **(A)** (PDB ID: 6U8A) and ZINC17988990 bound to the shallow portal site in TRPV5 **(B)** (PDB ID: 6PBE). Residues that belong to a specific structural domain are grouped in highlighted islands (green) and labeled. The analysis was performed using the LigPlot^+^ software (https://www.ebi.ac.uk/thornton-srv/software/LigPlus/).

Supplementary Figure S6 LigPlot^+^ analysis for the S1-S4 sites. LigPlot^+^ diagrams for 2-APB bound to the S1-S4 top site in TRPV3 **(A)** (PDB ID: 6DVZ), 2-APB-Br bound to the S1-S4 base site in TRPV6 **(B)** (PDB ID: 6D7V), osthole bound to the S1-S4 base site in TRPV3 **(C)** (PDB ID: 7RAS), and ZINC17988990 bound to the S1-S4 base site in TRPV5 **(D)** (PDB ID: 6PBE). Residues that belong to a specific structural domain are grouped in highlighted islands (green) and labeled. The analysis was performed using LigPlot^+^ software (https://www.ebi.ac.uk/thornton-srv/software/LigPlus/).

Supplementary Figure S7 LigPlot^+^ analysis for the ARD–TMD interface sites. LigPlot^+^ diagrams for 2-APB bound to the ARD-TMD linker site in TRPV3 **(A)** (PDB ID: 6DVZ), osthole bound to the ARD-TMD linker site in TRPV3 **(B)** (PDB ID: 7RAS), and PIP_2_ bound to the S2-S3 site in TRPV5 **(C)** (PDB ID: 6DMU). Residues that belong to a specific structural domain are grouped in highlighted islands (green) and labeled. The analysis was performed using LigPlot^+^ software (https://www.ebi.ac.uk/thornton-srv/software/LigPlus/).

Supplementary Figure S8 LigPlot^+^ analysis for the ARD site. LigPlot^+^ diagrams for ATP bound to TRPV1 **(A)** (PDB ID: 2PNN) and desthiobiotin bound to TRPV6 **(B)** (PDB ID: 5WO9). Residues that belong to a specific structural domain are grouped in highlighted islands (green) and labeled. The analysis was performed using LigPlot^+^ software (https://www.ebi.ac.uk/thornton-srv/software/LigPlus/).
