# Supplementary figures and images for: Ligand-Binding Sites in Vanilloid-Subtype TRP Channels
Source: Front Pharmacol. 2022 May 16;13:900623. doi: 10.3389/fphar.2022.900623 (PMC9149226; doi:10.3389/fphar.2022.900623)

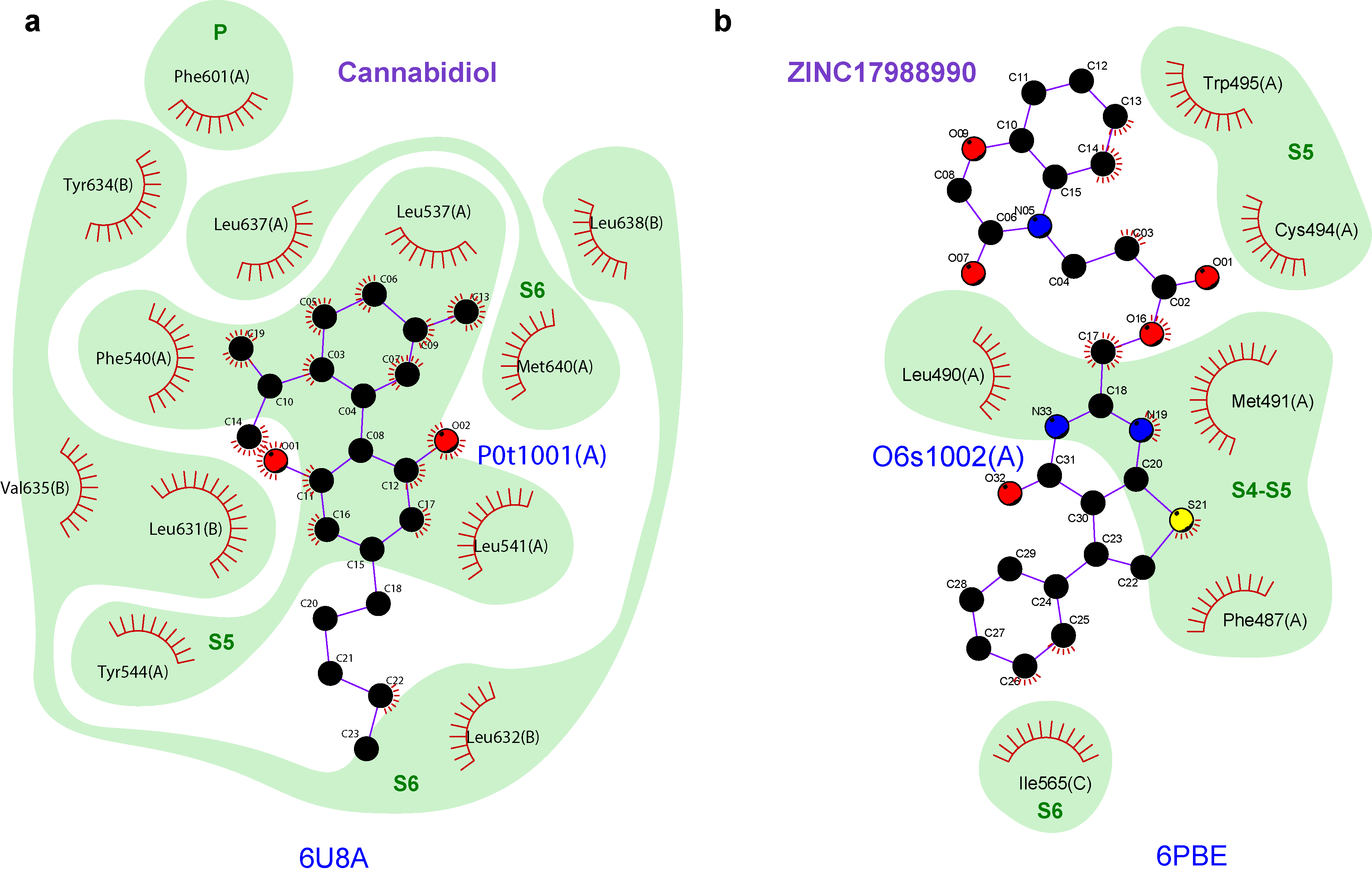

Supplement: Supplementary file 1 [file Image5.PNG]

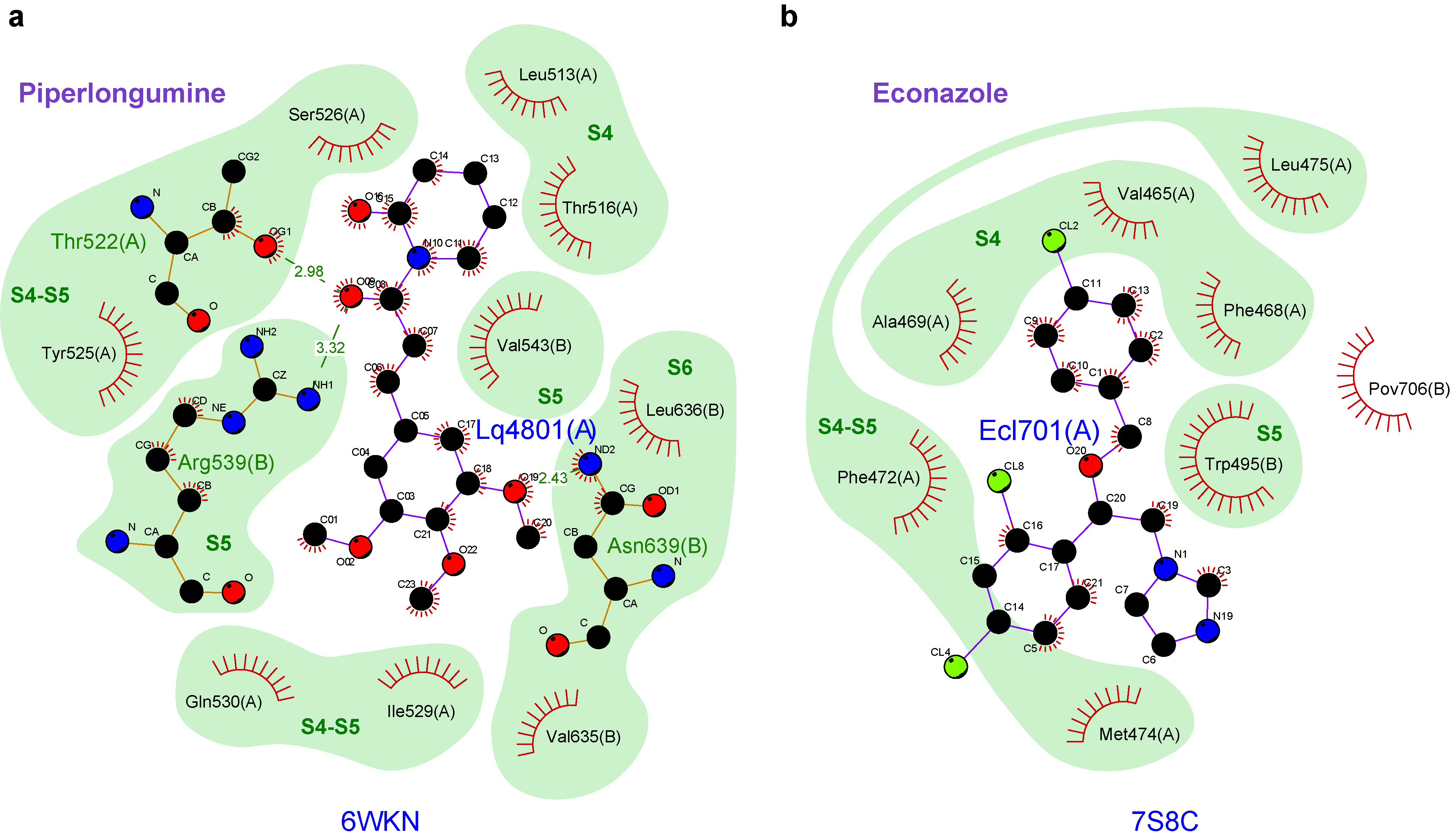

Supplement: Supplementary file 2 [file Image4.PNG]

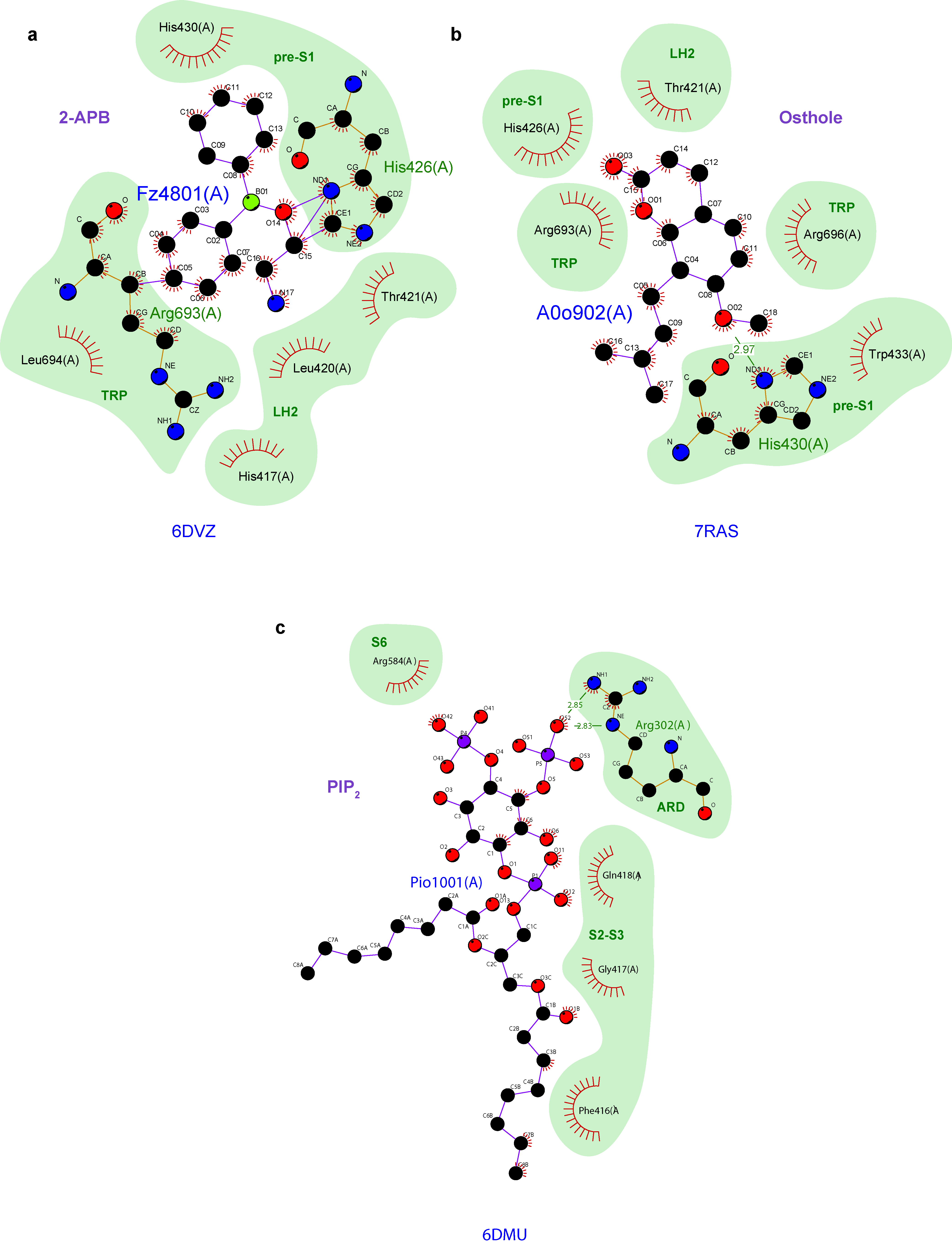

Supplement: Supplementary file 3 [file Image7.PNG]

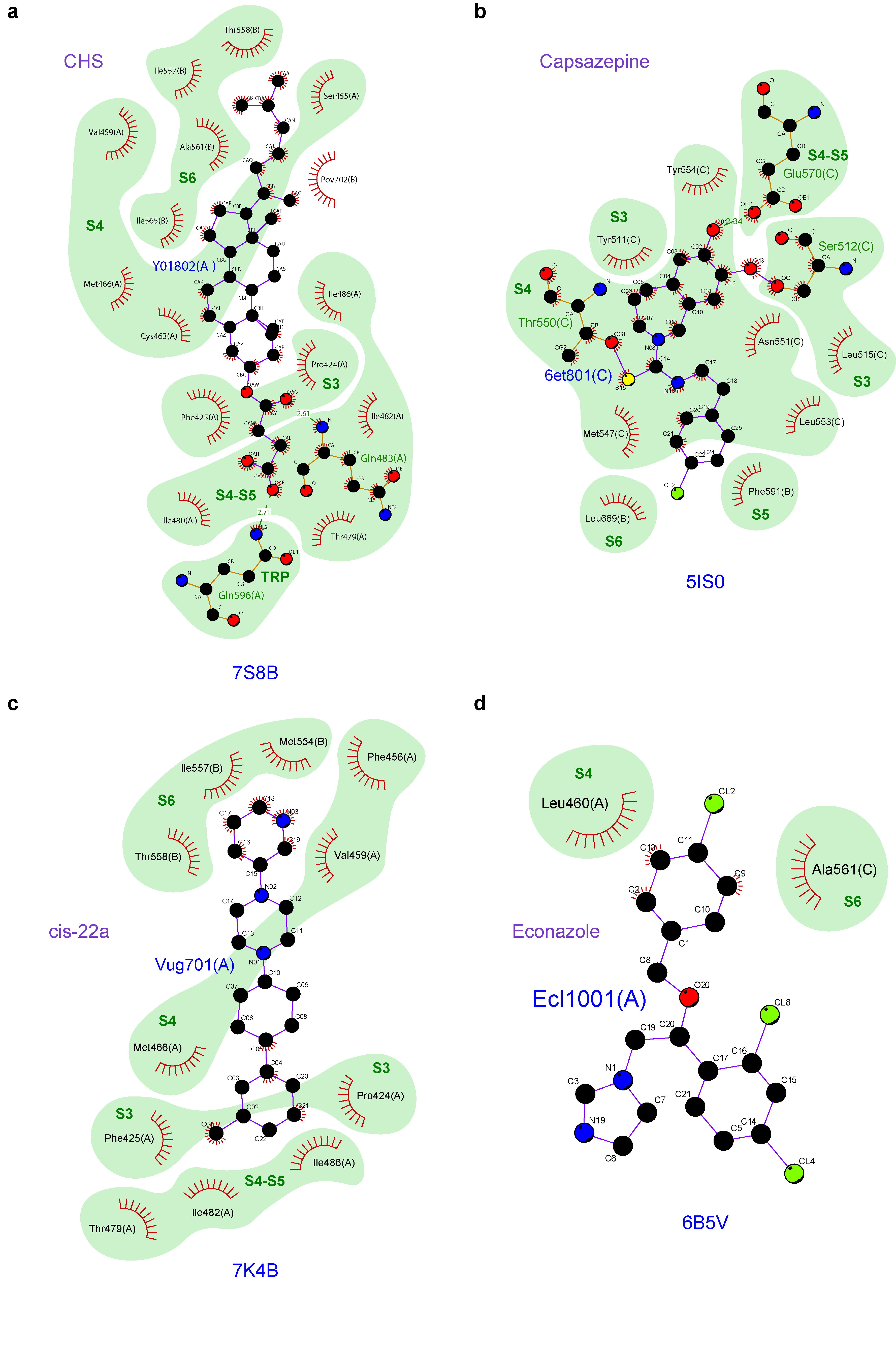

Supplement: Supplementary file 4 [file Image2.PNG]

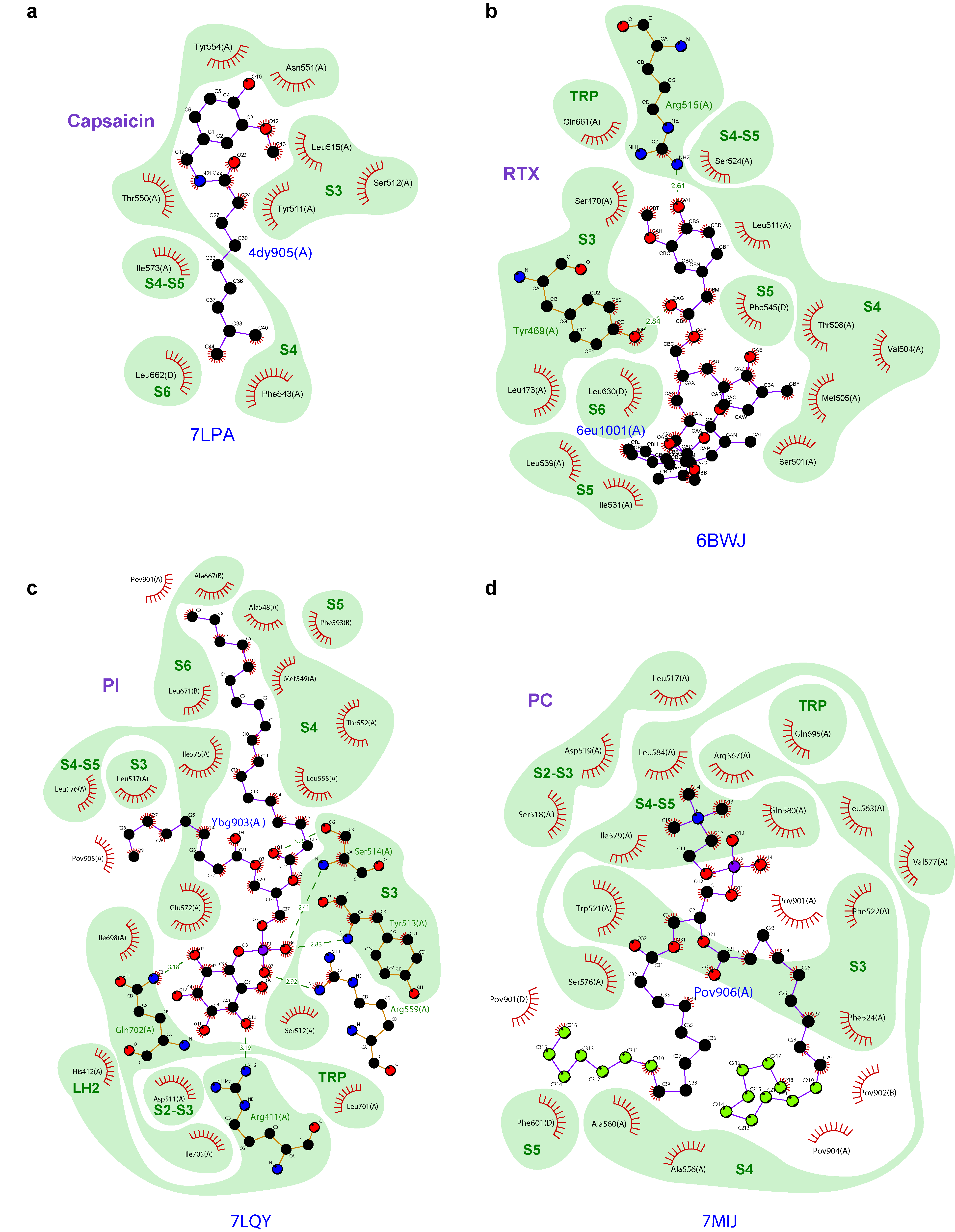

Supplement: Supplementary file 5 [file Image1.PNG]

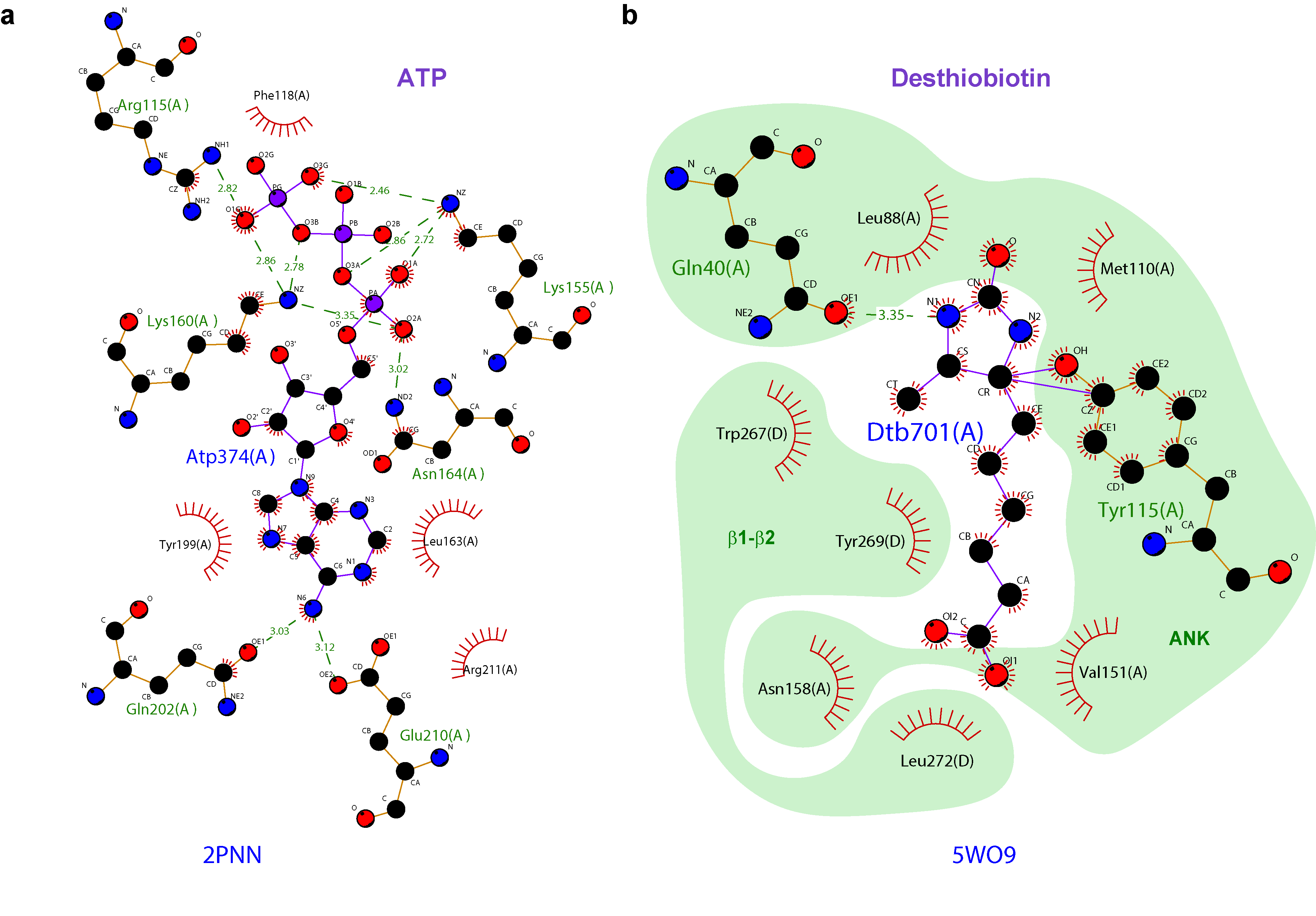

Supplement: Supplementary file 6 [file Image8.PNG]

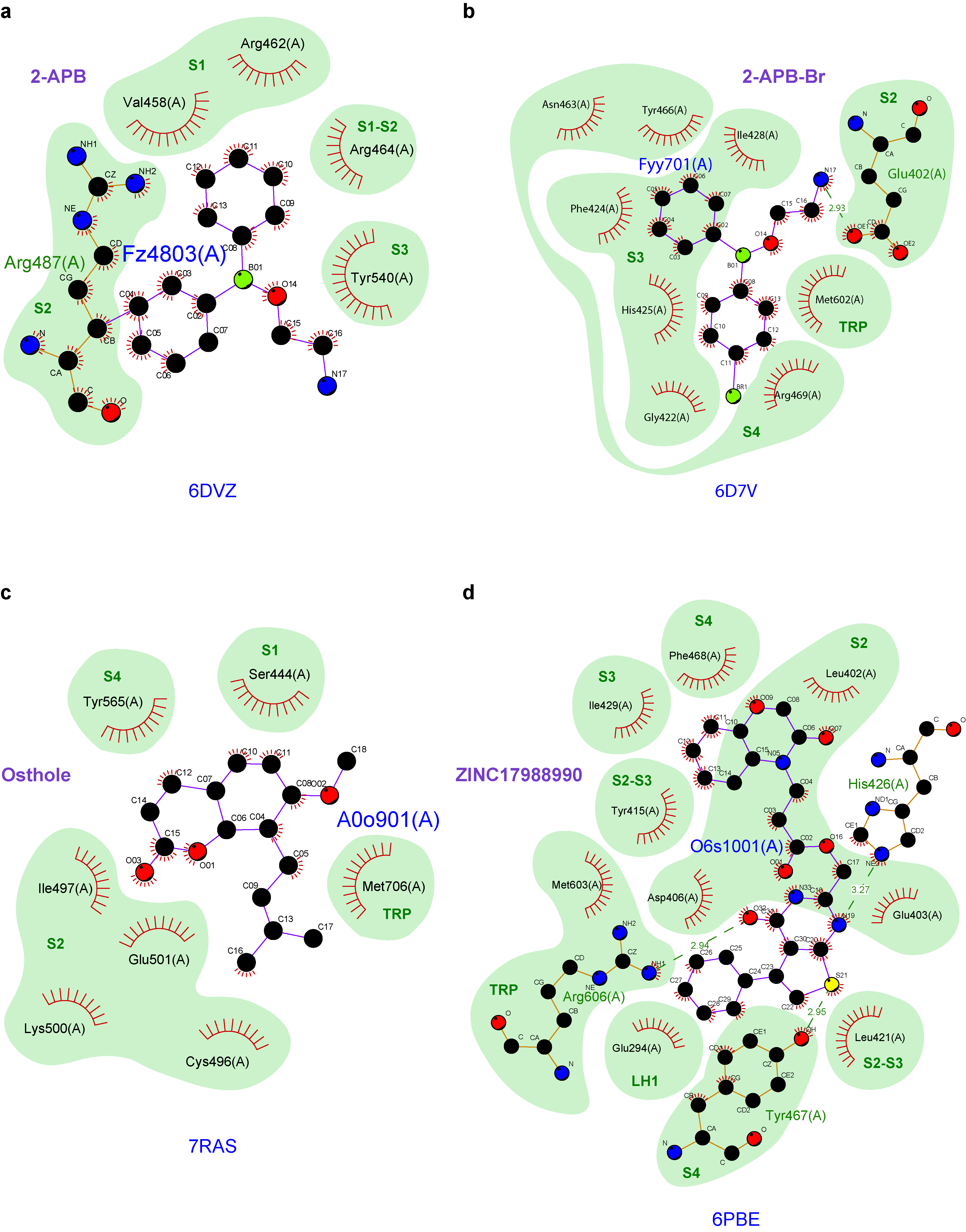

Supplement: Supplementary file 7 [file Image6.PNG]

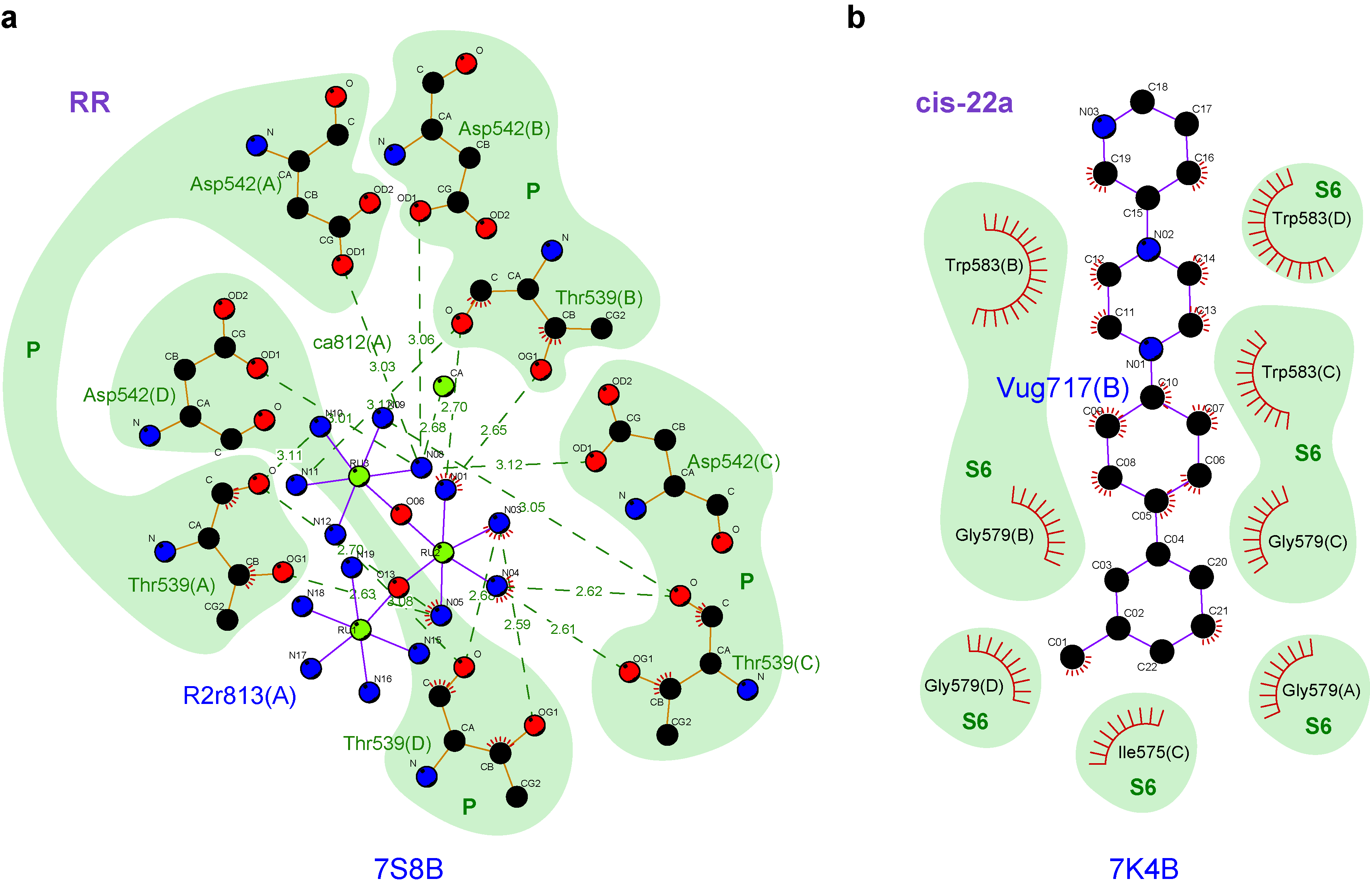

Supplement: Supplementary file 8 [file Image3.PNG]
